# Transformation Cascades in Iron Oxides: Quantitative Resolution of Sequential Precipitation Using the Reaction-Diffusion Framework

**DOI:** 10.1021/acsomega.5c11472

**Published:** 2026-02-05

**Authors:** Nour Abi Aad, Mazen Al-Ghoul

**Affiliations:** Department of Chemistry, 11238 American University of Beirut, Riad El-Solh 1107 2020, Beirut, Lebanon

## Abstract

Sequential phase transformations under transport limitation govern mineral formation, corrosion, and diffusion-driven synthesis, yet equilibrium phase diagrams and well-mixed experiments largely obscure transient intermediates, spatial segregation, and kinetic hierarchies. Here, we use precipitation–diffusion in 1.0 wt % agar hydrogels to resolve the transformation cascade of iron oxides as hydroxide (1.0–3.0 M NaOH) diffuses into Fe^2+^/Fe^3+^-loaded gels, producing three sharp, spatially separated fronts: yellow goethite (α-FeOOH), green rust (Fe^2+^–Fe^3+^ LDH), and black magnetite (Fe_3_O_4_). Quantitative tracking shows that front positions follow power-law kinetics, *d*_*i*_(*t*) = *α*_*i*_ *t* ^*β_i_*^, with *β_i_* spanning 0.39–0.56 and high goodness-of-fit (*R* ^2^ ≥ 0.97; typically >0.99) across all tested conditions. A coupled Stefan moving-boundary analysis links the parabolic front kinetics to phase-specific hydroxide consumption, yielding an alkalinity-demand hierarchy Λ_G_/Λ_GR_/Λ_M_ ≈ 1:1.3:1.9, which rationalizes both the transformation sequence and the progressive widening of the goethite region. Increasing outer hydroxide accelerates all fronts, whereas increasing total iron loading slows propagation; after 96 h, fronts typically penetrate 3–12 mm into the gel. Microscopy and spectroscopy support a solution-mediated dissolution–reprecipitation pathway, and Fe^2+^-rich conditions drive a transition from steady fronts to oscillatory Liesegang banding, demonstrating how diffusion–reaction balance controls both cascade formation and periodic precipitation.

## Graphical abstract

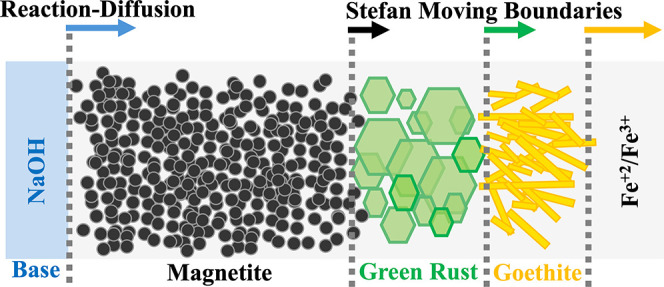

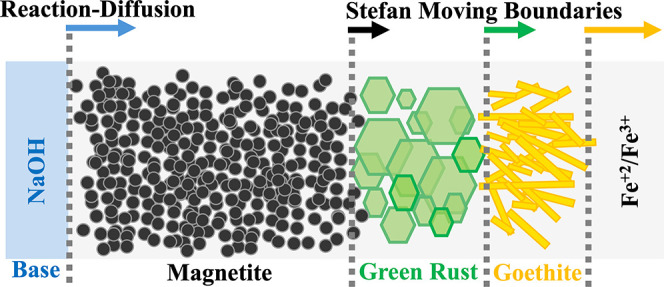

## 1 Introduction

Iron oxides and oxyhydroxides exhibit remarkable structural diversity and redox flexibility,^1^ which underlie their importance in catalysis, corrosion, environmental geochemistry, and emerging electronic and biomedical technologies.^2,3^ Among them, goethite (α-FeOOH), green rust (Fe^2+^–Fe^3+^ layered double hydroxide), and magnetite (Fe_3_O_4_) constitute a prototypical transformation cascade encompassing ferric, mixed-valent, and inverse-spinel phases. Their formation and interconversion through hydrolysis, redox exchange, and dissolution–reprecipitation govern iron cycling in both natural and synthetic environments, defining a spectrum of functional properties ranging from adsorption to magnetism.^4,5^

Iron-oxide nanoparticles are at the forefront of nanotechnology owing to their distinctive magnetic characteristics and chemical versatility. From metallurgy to biomedicine, they serve as platforms for catalysis, magnetic storage, environmental remediation, and targeted drug delivery.^6–9^ Magnetite is a mixed-valence ferrimagnetic oxide in which Fe^2+^ and Fe^3+^ cations occupy octahedral and tetrahedral sites of an inverse-spinel lattice,^10^ whereas goethite, an abundant ferric oxyhydroxide, exhibits surface hydroxyl groups that enable sorption and ion exchange.^11^ Despite their ubiquity, conventional syntheses of these phases (sol–gel,^12^ coprecipitation,^13,14^ hydrothermal growth^15^) proceed homogeneously and rapidly under well-stirred conditions, concealing the kinetic competition and intermediate phases that appear when transport is rate-limiting.

A particularly elusive member of this family is green rust (GR), a mixed-valent Fe^2+^–Fe^3+^ layered double hydroxide of general formula $\left\{{\left[{Fe}_{1-x}^{II}{Fe}_{x}^{III}{\left(OH\right)}_{2}\right]}^{x+}\cdot {\left[\left(x/n\right)A^{n-}\right]}^{x-}\cdot m\left[H_{2}O\right]\right\}$ .^16^ GR forms under mildly reducing, alkaline conditions by coprecipitation of Fe^2+^ and Fe^3+^ salts, yet it is metastable and readily oxidizes to more stable ferric oxyhydroxides. Its transient existence, together with its relevance to natural redox interfaces, motivates approaches that can stabilize and spatially confine this phase long enough for systematic study.

This challenge is directly addressed by the reaction–diffusion framework (RDF), wherein precipitation–diffusion (P–D) experiments in hydrogels provide unparalleled control over the transformation cascade. In our system, Fe^2+^/Fe^3+^ salts are immobilized within an agar matrix, while hydroxide from an overlying reservoir diffuses inward. The gel network is essential: it suppresses convection, enforces a steady diffusion gradient, and drastically slows precipitation kinetics, thereby making the sequential phase evolution directly observable.^17^ As hydroxide penetrates, a visually striking sequence of colored regions emerges: yellow (goethite), green (green rust), and black (magnetite), each corresponding to a discrete precipitation front. This setup does more than just reproduce known phases; it acts as a spatially resolved, transparent reactor within the RDF that uniquely stabilizes metastable green rust in situ and reveals the kinetic competition between phases that is otherwise concealed in conventional, well-stirred syntheses.^18^ The framework effectively freezes the transformation pathway in space, providing a direct visual record for quantitative analysis.

Beyond reproducing known phases, this framework stabilizes metastable GR in situ, enables controllable Liesegang banding through Fe^2+^/Fe^3+^ stoichiometry, and extends to MFe_2_O_4_ ferrites (M = Co, Ni, Cu, and Zn), positioning it as a versatile model for diffusion-driven phase evolution. The spatial segregation provided by the gel allows the extraction of phase-specific regions for structural, and spectroscopic characterization while preserving their formation history. Furthermore, RDF operates at room temperature and does not require glovebox-level anoxic handling.^19^ Although green rust is oxygen-sensitive, the phase cascade and front propagation in our sealed precipitation–diffusion geometry are primarily governed by the hydroxide diffusion field, and we verified that the dissolved oxygen in the gel remained low during the experiment (see Experimental Section). These observations rule out an oxygen gradient as the controlling driver of the spatially resolved G → GR → M sequence.

Because the color transitions between regions are microscopically abrupt (≤100 μm), each interface Γ_*i*_ where {*i* = G (goethite), GR (green rust), M (magnetite)}, can be treated as a Stefan moving boundary^20^ (Scheme 2) in which the inward diffusive flux of hydroxide balances its consumption at the reaction plane. In this framework, each front advances parabolically as *d*_*i*_(*t*) = *α*_*i*_ *t* ^1/2^, where the prefactor *α*_*i*_ depends on the hydroxide supply *c*_b_, effective diffusivity *D*_eff_, and a phase-specific alkalinity demand Λ_*i*_ that quantifies the hydroxide consumption per unit volume of precipitated solid. The resulting parabolic scaling *d*_*i*_∝*t* ^1/2^ signifies diffusion-limited front kinetics; the complete derivation and parameter extraction are provided in Section 3.3.1.

## 2 Experimental Section

### 2.1 Materials

Ferric chloride hexahydrate (FeCl_3_·6H_2_O, Strem Chemicals), ferrous ammonium sulfate hexahydrate [(NH_4_)_2_Fe­(SO_4_)_2_·6H_2_O, J.T. Baker], and sodium hydroxide (NaOH, VWR Chemicals) were used as received. Agar (BD Bacto Agar) served as the hydrogel matrix. All solutions were prepared with doubly distilled water (resistivity 18 MΩ cm). Agar was selected as the standard matrix because it provides a mechanically robust anticonvection scaffold over multiday experiments, enabling a stable one-dimensional diffusion field and reproducible precipitation fronts. The gel acts primarily as a physical transport medium; by suppressing convection and imposing a well-defined tortuosity, it renders the front motion sensitive to diffusion–reaction balance rather than mixing. We note that alternative biogels can modify effective diffusivity and may introduce additional chemical interactions (e.g., metal coordination in protein-based gels or fixed anionic groups in sulfated polysaccharides), which can influence local ion partitioning. For this reason, agar was used as a comparatively inert and stable baseline matrix for quantitative front-kinetics analysis.

### 2.2 Sample Preparation

A 1.0 wt % agar solution was prepared by dissolving agar in doubly distilled water under heating and constant stirring until complete dissolution. After cooling to ∼60 °C, FeCl_3_·6H_2_O and (NH_4_)_2_Fe­(SO_4_)_2_·6H_2_O were added to yield a homogeneous Fe^2+^/Fe^3+^ mixture with a molar ratio of 1:2. The hot solution (15.0 mL) was cast into glass test tubes (150 × 16 mm) and allowed to cool to room temperature and gel for at least 2 h. The resulting Fe^2+^/Fe^3+^ mixture in the gel had an initial pH of approximately 1. This acidic environment is established prior to the diffusion of the outer alkaline electrolyte, which further stabilizes the ferrous species against auto-oxidation prior to the start of the experiment. Subsequently, after gelation, 3.00 mL of 3.0 M NaOH solution (outer electrolyte) was carefully layered on top of the agar-iron gel (inner electrolyte) to establish a consistent gradient and to initiate the reaction–diffusion process.

After the addition of the alkaline reservoir, test tubes were immediately capped and sealed (e.g., with Parafilm) to minimize gas exchange during the multiday diffusion experiment. To assess whether oxygen could plausibly control the observed phase cascade, we monitored dissolved oxygen (DO) in the gel using an oxygen probe positioned in the bulk gel and near the advancing front using a dissolved O_2_ sensor (Orion 5 star pH meter–Thermo Scientific, 081010 Dissolved Oxygen Probe). DO values remained low (≈2 ppm, corresponding to ≈6 × 10^–5^ M under our conditions), indicating that any oxygen ingress was limited relative to the hydroxide supply. Accordingly, the spatial propagation of the precipitation fronts is interpreted as hydroxide-driven moving boundary behavior, rather than an artifact of the oxidation gradient.

The tubes were left undisturbed for 4 days to allow diffusion and precipitation fronts to develop. The goethite and magnetite regions were extracted, thoroughly washed with hot water (∼80 °C) to remove the agar gel and unreacted species, and then centrifuged to recover the precipitates for analysis. To prevent oxidation during handling, the green rust was rapidly extracted, immersed in liquid N_2_, and subsequently freeze-dried. The spatial location of each phase was reproducible within ±5% across triplicate experiments.

### 2.3 Material Characterization

Powder X-ray diffraction (PXRD) patterns were collected on a Bruker D8 Discover diffractometer using Cu Kα radiation (λ = 1.5408 Å) at 40 kV and 40 mA, with 2*θ* ranging from 10° to 70° at a scan rate of 5° min^–1^. UV–Vis diffuse reflectance spectra were recorded on a PerkinElmer LAMBDA 1050+ UV/vis/NIR spectrophotometer. Scanning electron microscopy (SEM) images were obtained with a Tescan MIRA3 LMU equipped with an Oxford energy-dispersive X-ray (EDX) detector for elemental analysis, operated at 15 kV. Attenuated total reflectance Fourier transform infrared (ATR-FTIR) spectra were collected on a Bruker Tensor 27 spectrometer with a diamond ATR module. Additionally, the N_2_ adsorption–desorption isotherms were measured at 77 K using 3Flex Micromeritics. Before measurement, magnetite and goethite were degassed under vacuum at 90 °C overnight; however, to preserve the metastable green rust phase, the GR sample was not subjected to the overnight degassing.

### 2.4 Digital Image Analysis and Front Tracking

To quantify the reaction front kinetics, the time-lapse video recordings were analyzed using a custom MATLAB script. The time-dependent position of the precipitation front was extracted by monitoring the optical intensity profiles along the central vertical axis of each reactor tube. The blue color channel for the green rust region (and the red color channel for the goethite) was specifically isolated for analysis as it provided the optimal signal-to-noise ratio for distinguishing the reaction boundary. The interface position was determined frame by frame using the findpeaks function, which identified the most prominent intensity peak along the longitudinal axis. To ensure robust detection and eliminate artifacts, a minimum peak height threshold was set at 50% of the maximum channel intensity, and the upper meniscus region (approximately the top 20 pixels) was excluded from the analysis window. The resulting temporal evolution of the peak locations was exported to generate the distance-versus-time plots (*d* vs *t*) presented in Figure 1. The same method was also used to extract the spacing coefficient, *Q*, of the Liesegang bands.

**Figure 1:**
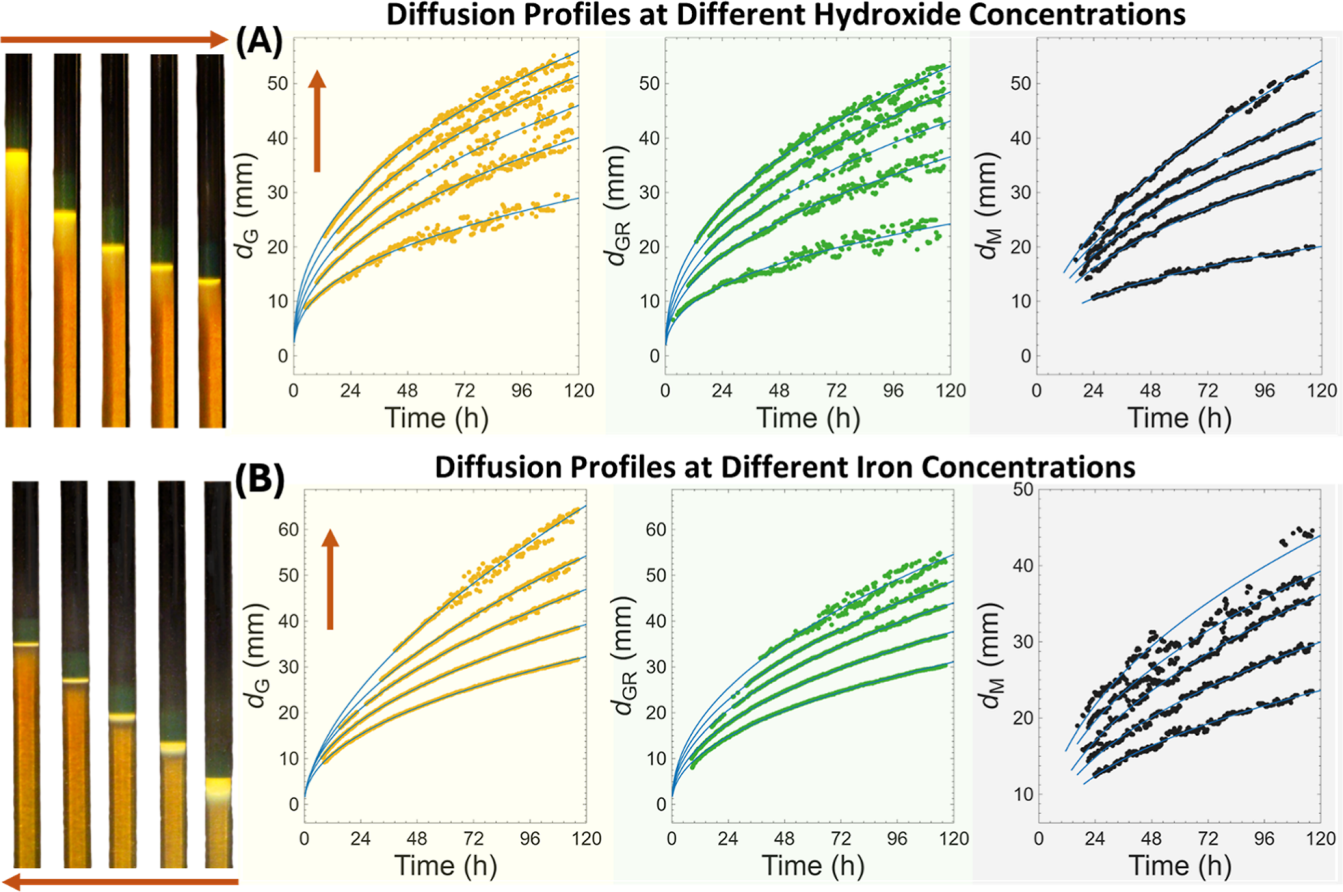
Reaction–diffusion fronts in Fe^2+^/Fe^3+^-loaded agar hydrogels under varying hydroxide and iron concentrations. (A) Hydrogels (1.0 wt %) containing fixed [Fe^2+^] = 0.10 M and [Fe^3+^] = 0.20 M were exposed to outer electrolytes of increasing [NaOH] (1.0–3.0 M; arrow), corresponding to an increasing OH^–^ supply. Higher hydroxide concentration accelerates the advance of the yellow, green, and black regions corresponding to goethite (α-FeOOH), green rust (Fe^2+^–Fe^3+^ LDH), and magnetite (Fe_3_O_4_), respectively, while preserving the diffusion-limited parabolic dependence of front position on time *d*_*i*_ = α*t* ^1/2^. (B) At fixed [NaOH] = 3.0 M, hydrogels containing decreasing total iron concentrations ([Fe^2+^]/[ Fe^3+^] = 0.20/0.40 to 0.04/0.08 M; arrow) exhibit faster front propagation with lower Fe content, consistent with a smaller hydroxide-consumption coefficient (Λ_i_) in the Stefan moving-boundary framework. In both panels, symbols represent experimental data, and solid curves represent power-law fits for *d*_G_(*t*), *d*_GR_(*t*), and *d*_M_(*t*); the fitting parameters are summarized in the Supporting Information, confirming that diffusion-controlled propagation is governed by hydroxide supply and interfacial reactivity.

**Scheme 1:**
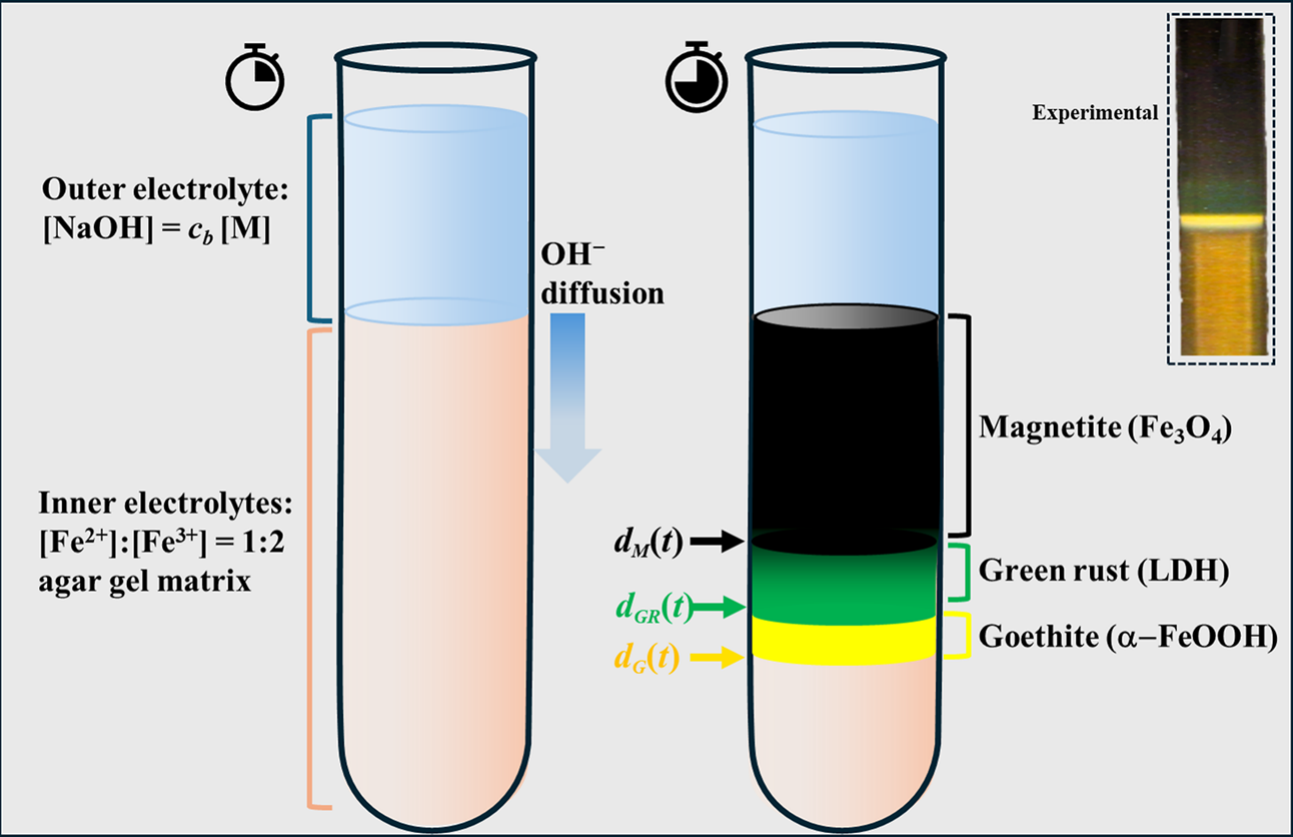
Schematic illustration of the reaction–diffusion–driven precipitation sequence of iron oxides in an agar hydrogel^a^

a Upon the diffusion of an outer electrolyte (NaOH) with a concentration (*c*_b_) into a 1.0 wt % agar matrix containing mixed Fe^2+^/Fe^3+^ ions (molar ratio 1:2), a self-organized precipitation front develops and advances with time (The resulting experimental pattern is shown above). The front delineates three distinct colored regions: a yellow region of goethite (α-FeOOH) at the leading edge, a green region of green rust (Fe^2+^–Fe^3+^ layered double hydroxide) in the intermediate zone, and a black region of magnetite (Fe_3_O_4_) corresponding to the most reduced and thermodynamically stable phase. The spatial segregation of these regions, marked by the time-dependent positions *d*_G_(*t*), *d*_GR_(*t*), and *d*_M_(*t*) provides a direct visual record of the sequential transformation from ferric oxyhydroxide to mixed-valence hydroxide to spinel oxide under diffusive base supply.

### 2.5 Verification of Fe­(II) Stability

The stability of Fe^2+^ within the gel matrix during the diffusion experiment was validated using a thiocyanate colorimetric test. Potassium thiocyanate (KSCN) was incorporated into the gel matrix as a selective indicator for Fe^3+^ (forming a red complex). Control experiments demonstrated that while exposed Fe^2+^ gels slowly oxidize over time, the application of the outer hydroxide layer results in the rapid formation of an interfacial precipitate. This surface layer acts as a passivation barrier, preventing oxygen diffusion into the bulk gel. Consequently, the bulk Fe^2+^ ahead of the reaction front remains stable (indicated by the absence of the red Fe^3+^-SCN complex throughout the time scale of the precipitation experiments (Figure S1).

## 3 Results and Discussion

### 3.1 Macroscopic Precipitation-Diffusion Patterns and Phase Identification

Layering an alkaline solution above an Fe^2+^/Fe^3+^-loaded agar hydrogel produces a visually striking sequence of spatially separated color regions that evolve (refer to Scheme 1). A thin yellow region forms first near the gel–solution interface, followed by the emergence of a green region and, subsequently, a broader black region deeper within the gel. These correspond respectively to goethite (α-FeOOH), green rust (Fe^2+^–Fe^3+^ layered double hydroxide), and magnetite (Fe_3_O_4_), as later confirmed by XRD and vibrational spectroscopy (see Figure 2A,B). The regions are sharply delineated, both macroscopically and microscopically (color transitions occur within a few hundred microns), allowing each interface to be treated as a distinct precipitation–diffusion front advancing under the supply of hydroxide ions from the outer solution.

**Figure 2:**
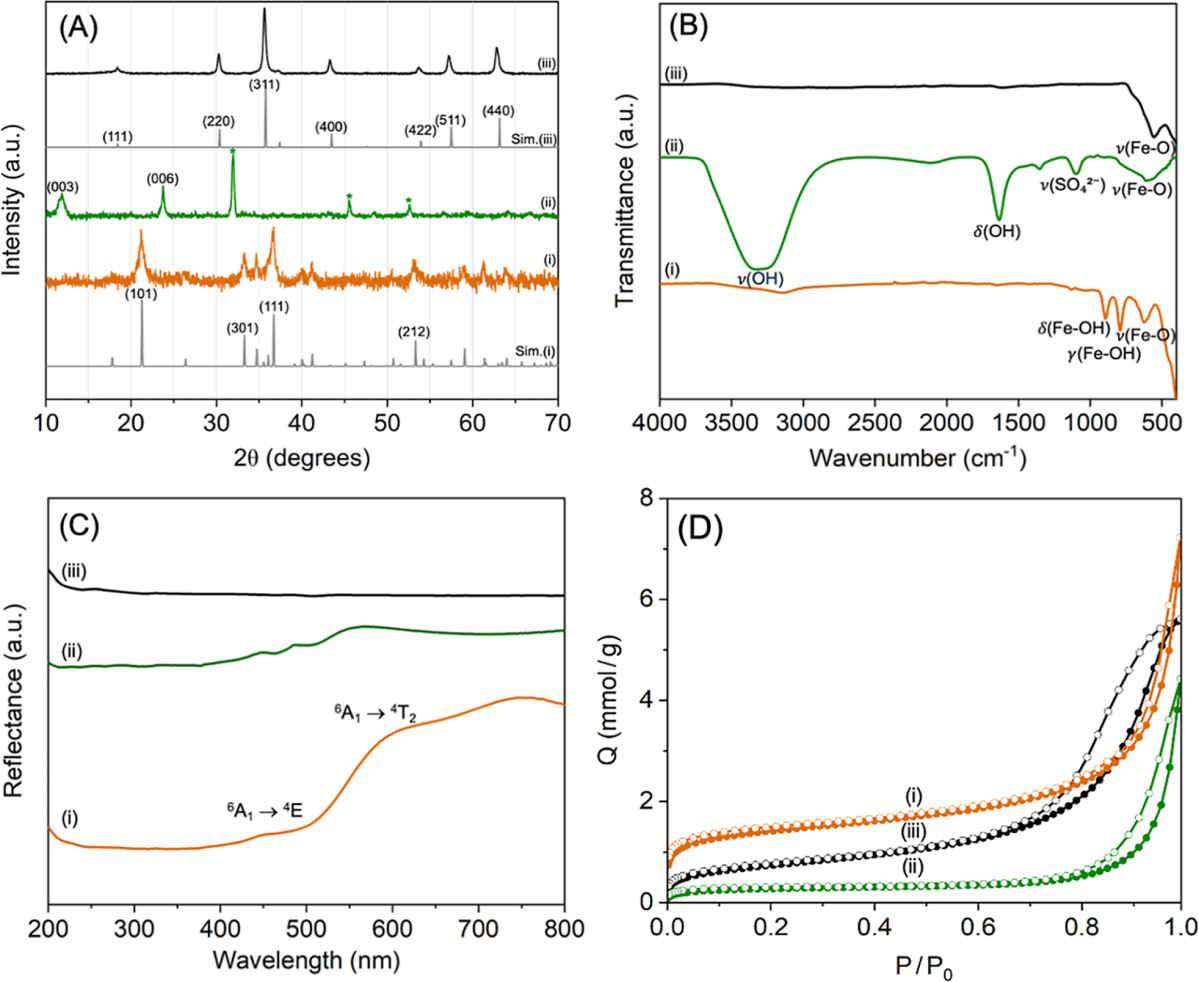
(A) Powder X-ray diffraction (PXRD) patterns of iron oxide phases obtained from the precipitation–diffusion system: (i) yellow leading region corresponding to goethite (α-FeOOH), (ii) intermediate green region assigned to green rust (Fe^2+^–Fe^3+^ layered double hydroxide), and (iii) black region identified as magnetite (Fe_3_O_4_), simulated reference patterns are shown below each experimental pattern: Sim (i) corresponds to goethite (JCPDS 29-0713) and Sim (iii) corresponds to magnetite (JCPDS 19-0629). Minor peaks marked with asterisks (*) in pattern (ii) originate from residual NaCl; (B) ATR-FTIR transmittance spectra of the three phases; (C) UV–Vis diffuse reflectance spectra of (i) goethite (α-FeOOH), showing weak spin-forbidden transitions, (ii) green rust, displaying a broad intervalence charge-transfer absorption; and (iii) magnetite, characterized by nearly featureless broadband absorption across the visible region. These optical signatures directly correlate with the distinct yellow, green, and black precipitation regions observed in the gel system; and (D) Nitrogen adsorption–desorption isotherm of goethite (iii), green rust (ii), and magnetite (i) at 77 K, showing the quantity adsorbed and desorbed (mmol g^–1^) as a function of relative pressure (*P*/*P*_0_).

The overall sequence is robust and reproducible across a wide range of compositions. At a fixed inner Fe^2+^/Fe^3+^ ratio (1:2) and increasing outer hydroxide concentration (1.0–3.0 M NaOH), all three fronts propagate faster, producing deeper and more sharply resolved regions (Figure 1A). Conversely, at fixed outer [NaOH] = 3.0 M, decreasing the total Fe^2+^ + Fe^3+^ concentration (0.60 to 0.12 M) yields the same ordered sequence but with greater front velocities and reduced region intensities (Figure 1B). These observations indicate that the supply of hydroxide and total iron loading jointly determine the advancement rate of each interface: the hydroxide flux accelerates the fronts, whereas a higher inner iron concentration slows them by increasing the local demand for hydroxide. After 96 h, fronts typically lie 3–12 mm below the surface (0.5–3 mm day^–1^), depending on the concentration of NaOH and the Fe loading. The regions formation and transformation, accompanied by a consequent color change from yellow to green to black, are clearly visible in Videos S1 and S2.

A salient feature is that the yellow goethite region widens with time, demonstrating that its rate of formation exceeds its rate of consumption in the subsequent goethite → green rust transformation. The persistence of a distinct goethite zone suggests that the leading and trailing boundaries, associated respectively with α-FeOOH precipitation and its reductive replacement, move at different speeds. As shown later, this difference translates into a measurable offset between the fitted prefactors *α*_lead_ and *α*_trail_ in the parabolic law *d* = *αt* ^1/2^ and *w*(*t*) = (*α*_lead_–*α*_trail_)*t* ^1/2^.

The observed dependencies on hydroxide and iron concentration already hint at diffusion-limited moving-boundary behavior, as predicted by the Stefan formulation introduced previously. Increasing the outer [OH^–^] raises the boundary concentration *c*_b_, thereby enhancing the diffusive flux and the prefactor *α*_*i*_, while decreasing the total Fe content reduces the reactive sink strength Λ and produces the same effect. The apparent invariance of the time exponent (*β* ≈ 0.5) across all conditions reinforces the diffusion-controlled nature of the process. The macroscopic color boundaries, therefore, map directly onto the self-similar diffusion fronts of the Stefan problem, allowing for the quantitative extraction of kinetic parameters in the following section. On a microscopic level, crystallite size trends were analyzed as a function of initial hydroxide and iron concentration by comparing the same phase region from the experiments conducted under systematically varied conditions. The study of particle size for a specific phase as a function of outer and inner electrolyte concentration is supported by the transformation mechanism, since each phase forms via a sharp moving front driven by dissolution and reprecipitation, rather than by gradual precipitation along a fixed gradient. Scanning electron microscopy, as displayed in Figures S2–S4, reveals the growth mechanism of the particles with increasing inner iron concentration. The spherical magnetite particles undergo an isotropic growth resulting in the coarsening of the smaller particles, whereas the acicular crystals exhibited an anisotropic growth, elongating along the *c*-axis ([001] direction). The decrease in outer hydroxide concentration, while keeping the inner iron concentration fixed, waned the nucleation rate, resulting in lower supersaturation, which promoted the growth of larger particles.

It is important to emphasize that the crystallite-size trends shown in Figures S5–S7 do not represent spatial variations along a single diffusion column. Instead, they compare the size of a given iron oxide phase extracted from equivalent precipitation fronts formed in separate experiments conducted at different outer hydroxide concentrations. In this reaction–diffusion system, each phase forms at a sharp moving interface via a dissolution–reprecipitation mechanism, rather than by progressive nucleation and growth along a continuous supersaturation gradient. Consequently, the crystallite size within a given colored region reflects the local supersaturation at the formation front imposed by the hydroxide supply, not the position within the gel. Higher outer [NaOH] increases the local supersaturation at the reaction front, leading to higher nucleation density and therefore smaller final crystallites, whereas lower [NaOH] favors fewer nuclei and allows for slightly larger crystallite growth. This behavior is fully consistent with classical nucleation theory and does not contradict established Liesegang observations, which describe spatial gradients within a single experiment.

Moreover, varying the agar concentration modifies the front behavior without altering the phase sequence. Denser gels (≥1.5 wt %) slow hydroxide penetration, resulting in narrower regions and shorter overall propagation distances (Figure S8). This reflects a decrease in the effective hydroxide diffusivity *D*_eff_[m^2^ s^–1^] with increasing matrix tortuosity, as expected for diffusion in polymer networks. The resulting trends: slower, thinner, and sharper fronts at higher agar content, further confirm that the precipitation–diffusion dynamics are governed primarily by transport rather than by intrinsic solid-state kinetics. The impact of gel concentration on particle size reveals that increasing the gel percentage leads to inhibition of growth. Although the gel creates a stable, convection-free matrix, it also physically limits crystal growth due to a reduction in pore size with an increase in nucleation site density.^21^ At a higher gel percentage, the gel network increases the viscosity of the medium, thereby restricting the coalescence of particles by limiting the diffusion of ions. As demonstrated by the SEM images in Figure S8, the particles exhibited discernible but minor changes as the size of magnetite nanoparticles was marginally reduced across the higher concentrations. This indicates that the gel matrix primarily acted as a limiting network that tapered with the growth of the nanoparticles.

Finally, the influence of the basic medium was studied by screening different alkaline sources: 3.0 M sodium hydroxide (NaOH), 3.0 M potassium hydroxide (KOH), and ∼13 M ammonia (NH_3_, 25%). The three characteristic phases were observed in all cases, confirming the invariability of the reaction pathway, regardless of the alkali source. As observed in Figure S9, the ammonium hydroxide base resulted in notably faster diffusion fronts, while KOH yielded a pattern almost identical to that of NaOH. While the three solutions are strongly alkaline, they differ substantially in their bulk hydroxide ion activity. This difference in concentration directly governs the diffusive hydroxide flux into the gel, leading to distinct front-propagation kinetics in the system. The faster diffusion observed for the ammonium hydroxide is primarily due to the large reservoir of hydroxide ions provided by the high concentration of ammonia, which leads to a continuous replenishing of the OH^–^ ions at the interface. At the same molarity, KOH and NaOH have nearly identical ionic strengths, pH values, and driving forces (hydroxide concentrations), resulting in similar diffusion and distinct phase formation. Scanning electron microscopy (SEM) analysis confirmed (refer to Figure S10) that the identity of the base did not affect the final morphology of the three phases despite the differing diffusion behavior. In all three cases, the same crystal habits were observed: defined hexagonal green rust sheets, spherical magnetite nanoparticles, and acicular goethite crystals. In this study, we proceeded with NaOH as a representative outer electrolyte to ensure a consistent basis for comparison.

Taken together, the macroscopic observations demonstrate a consistent, diffusion-limited advance of three coupled precipitation fronts whose relative speeds depend systematically on hydroxide supply, reactive sink strength, and gel permeability. These findings provide the experimental foundation for the quantitative front-kinetics and Stefan analysis presented next. The optical sharpness of the yellow–green–black transitions (≤100–300 μm) motivates a moving-boundary description in which each colored interface is treated as a Stefan front.

### 3.2 Spectroscopic Characterization of Iron Oxide Phases

To confirm the assignment of the yellow, green, and black precipitation–diffusion regions to distinct iron oxide phases, each region was excised from the gels, purified, and subjected to PXRD, SEM, ATR-FTIR, and UV–Vis diffuse reflectance spectroscopy. These complementary analyses not only established the identity, morphology, and electronic structure of the products but also clarified how macroscopic trends, such as suppression or sharpening of regions, map onto the underlying phases. The PXRD patterns of the three regions shown in Figure 2A directly support their identification as goethite, green rust, and magnetite. The yellow region showed broadened reflections of orthorhombic α-FeOOH with characteristic peaks at 2*θ* = 21.4°, 33.5°, 36.7°, and 53.2° (JCPDS 29-0713). The green region displayed the diagnostic basal reflections of layered double hydroxides at (003) and (006), the minor peaks marked with asterisks (*) correspond to residual NaCl, which was not removed to preserve the metastable green rust phase. The black region gave the expected cubic spinel reflections of Fe_3_O_4_ at 2*θ* = 30.5°, 35.5°, 43.4°, 53.6°, and 62.8° (JCPDS 19-0629).^22^ The crystallite sizes estimated from peak broadening (∼20 nm) matched the nanoscale morphologies seen by SEM. The diffraction peaks confirm the sequential phase transformation from ferric oxyhydroxide to mixed-valent hydroxide to spinel oxide.

ATR-FTIR spectra in Figure 2B confirmed the vibrational signatures of each phase and their structural evolution. Goethite exhibited δ­(Fe–OH) and γ­(Fe–OH) modes at 892 and 794 cm^–1^ and Fe–O stretches below 600 cm^–1^. Green rust displayed a broad vibration OH stretch (∼3400 cm^–1^), δ­(OH) bending at ∼1630 cm^–1^, a sulfate interlayer vibration at ∼1100 cm^–1^, and Fe–O vibrations below 600 cm^–1^. The presence of intercalated carbonate ions in the layered green rust was also confirmed by the presence of a stretching vibration at 1353 cm^–1^. Magnetite showed a sharp Fe–O stretch mode at ∼570 cm^–1^. The ATR-FTIR confirms structural evolution: goethite’s Fe–OH deformation (892 cm^–1^) replaced by GR’s sulfate interlayer vibration (1100 cm^–1^), then magnetite’s sharp Fe–O stretch (570 cm^–1^).

Optical spectra, presented in Figure 2C, directly explain the color of the regions visible in the gel. Goethite exhibited weak spin-forbidden Fe^3+^ ligand-field d–d transitions at ∼480 and 650 nm, which contributed to its yellow appearance. Green rust exhibited a broad intervalence Fe^2+^/Fe^3+^ charge-transfer absorption peak centered at approximately 560 nm, which is responsible for its characteristic green hue.^23^ Magnetite showed broadband absorption across the visible region, consistent with its black color. The spectral signatures thus validate the direct link between region color and phase identity, explaining why subtle changes in the Fe^2+^/Fe^3+^ ratio or OH^–^ supply led to the visible darkening of the green region or its replacement by black magnetite.

Nitrogen adsorption–desorption isotherms performed at 77 K in Figure 2D, along with a BET analysis, reveal distinct porosity evolution (Table S7). PXRD patterns collected after BET outgassing (90 °C, N_2_) confirm that the activation procedure did not alter the phase composition (see Figure S11). The goethite isotherm exhibits a Type IV profile, typical of mesoporous adsorbents, with a narrow H3-type hysteresis loop. The BJH (Barrett–Joyner–Halenda) analysis shows a mesopore size distribution with a pore width of around 17.1 nm. The magnetite isotherm exhibits a Type IV isotherm with the existence of an H1 type hysteresis loop closing near *P*/*P*_0_ ≈ 0.43, indicative of agglomerates of generally uniform spheres. The green rust isotherm resulted in a Type IV profile with a H3-type hysteresis characteristic of aggregates of plate-like particles. The BET analysis links the morphology to the textural properties, confirming that the magnetite spherical nanoparticles derive their porosity from their highly regular packing. In contrast, the hexagonal green rust platelets yielded diagnostic slit-shaped pores generated by the stacking of the two-dimensional sheets, and the porosity in the needle-shaped goethite particles arose from the interconnected voids within the aggregated bundles.

### 3.3 Stefan Analysis of Front Kinetics

#### 3.3.1 Theoretical Framework

The theoretical framework developed here provides the physical basis for the parabolic scaling observed experimentally and allows the extraction of the key parameters *α*_*i*_ and Λ_*i*_ from the measured front positions *d*_*i*_(*t*). Quantitative tracking of the color boundaries yielded the front positions *d*_*i*_(*t*) (mm) that follow parabolic power laws in time, *t*(*h*),*d*_*i*_(*t*) = *α*_*i*_ *t* ^*β_i_*^ (*R* ^2^ > 0.99, Tables S1–S6). While ideal diffusion-limited Stefan fronts exhibit *β* = 0.5, the experimentally extracted exponents in the present system span the range *β* ≈ 0.40–0.54. These modest deviations reflect microstructure-mediated modifications of the effective hydroxide diffusivity caused by phase-specific precipitation morphologies, rather than a breakdown of diffusion control. Dense precipitates (magnetite) locally hinder transport and yield *β* < 0.5, whereas porous precipitates formed under high hydroxide flux slightly enhance transport and yield *β* ≥ 0.5. This deviation is consistent with SEM-observed porosity changes (Section 3.6). In all cases, the persistence of single-power-law scaling with *R* ^2^ > 0.99 and the systematic dependence of the prefactor on hydroxide supply confirm diffusion-limited moving-boundary behavior. The precipitation–diffusion system can be described by a one-dimensional Stefan moving-boundary problem for hydroxide diffusion into the Fe^2+^/Fe^3+^ loaded gel. Hydroxide diffuses from the outer reservoir (concentration *c*_b)_ into a semi-infinite gel (effective diffusivity *D*_eff_), forming sharp reaction planes at positions *d*_*i*_(*t*) where {*i* = G (goethite), GR (green rust), M (magnetite)}.

To validate the application of the Stefan moving-boundary model, which assumes quasi–discontinuous interfaces, we quantified the spatial width of the reaction zones using high-resolution optical microscopy. The transition region (*δ*) between phases (e.g., goethite to green rust) exhibits a measured width of approximately 370 μm (Figure S12).

Given that the macroscopic diffusion fronts propagate over distances of 20–60 mm, the reaction zone width represents less than 2% of the total diffusion domain (δ/*L*). This scale analysis confirms that the interfaces are sufficiently sharp to be treated as mathematical boundaries where the diffusive flux is balanced by localized consumption. Furthermore, the abrupt morphological transitions observed in SEM (Figure 4) corroborate that the dissolution–reprecipitation mechanism is spatially confined to this narrow interfacial window.

Therefore, we formulate the three-phase precipitation-diffusion system as a coupled moving-boundary problem with three consecutive reaction fronts (Scheme 2). The model describes the diffusion of hydroxide from an alkaline reservoir into a Fe^2+^/Fe^3+^-loaded gel, with the sequential precipitation of goethite (α-FeOOH), green rust (Fe^2+^–Fe^3+^ LDH), and magnetite (Fe_3_O_4_).

**Scheme 2:**
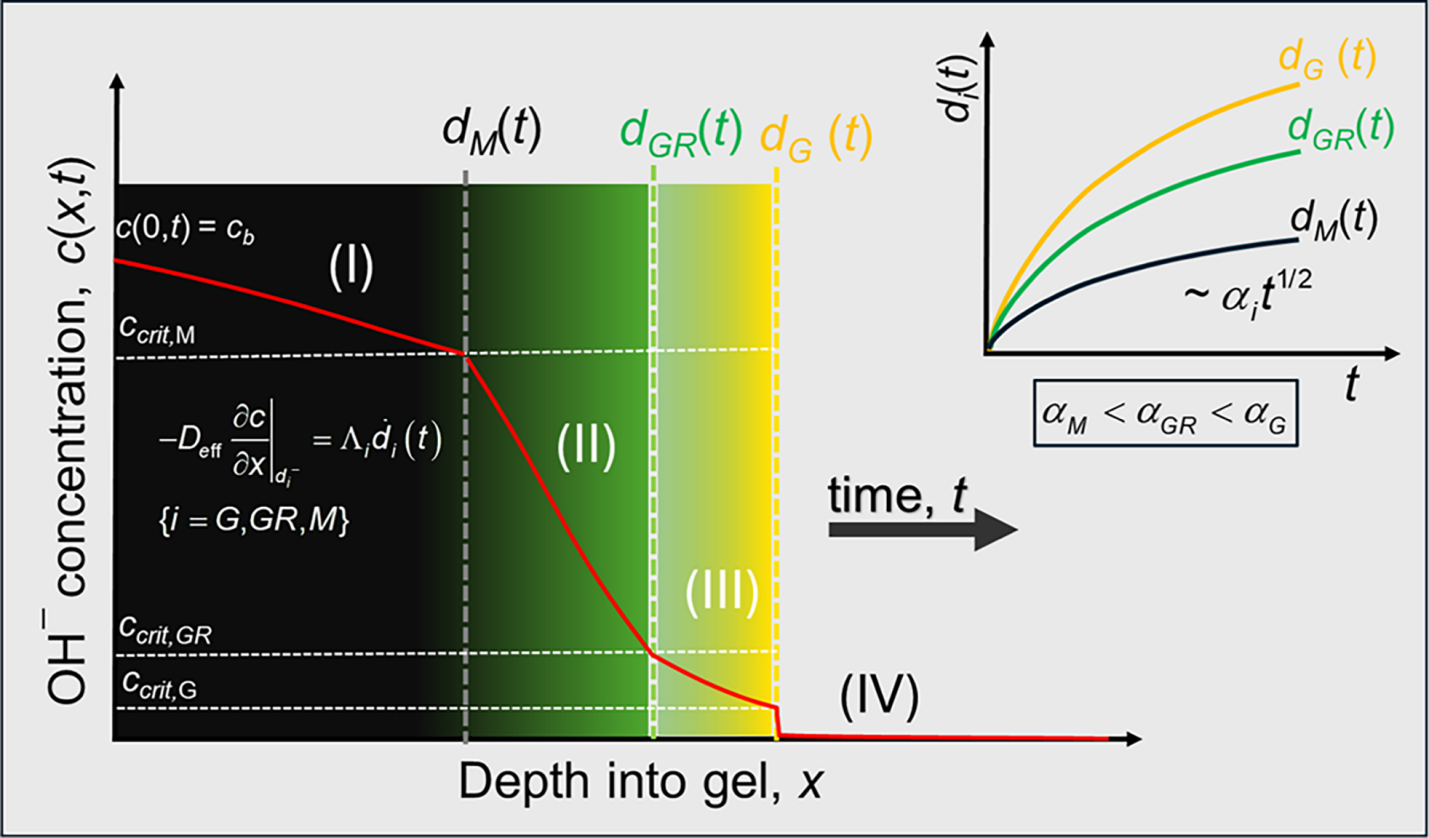
Stefan Moving-Boundary Framework for Sequential Iron Oxide Precipitation^a^

a Schematic representation of the one-dimensional reaction-diffusion system showing the hydroxide concentration profile and advancing precipitation fronts (See eqs 4 –6 for the analytical solutions). Hydroxide ions (OH^–^) diffuse inward from an alkaline reservoir maintained at constant concentration *c*(*x*,*t*) = *c*_*b*_, penetrating into an Fe^2+^/Fe^3+^-loaded agar gel with effective diffusivity *D*_eff_. The system evolves through four distinct regions separated by three moving precipitation fronts: Region I (Magnetite Zone, 0 ≤ *x* ≤ *d*_M_(*t*)): black magnetite (Fe_3_O_4_) precipitates where hydroxide concentration exceeds the highest critical threshold *c*_crit,M_. This innermost region experiences the strongest hydroxide flux. Region II (Green rust zone**,** *d*_M_(*t*) ≤ *x* ≤*d*_GR_(*t*)): Green rust (Fe^2+^–Fe^3+^ LDH) forms at intermediate hydroxide concentrations *c*_crit,GR_, representing the metastable mixed-valent phase. Region III (Goethite zone, *d*_GR_(*t*) ≤ *x* ≤ *d*_G_(*t*)): yellow goethite (α-FeOOH) precipitates at the leading edge where hydroxide concentration first exceeds the lowest threshold *c*_crit,G_. Region IV (Unreacted Zone, *x* > *d*_G_(*t*)): No precipitation occurs where hydroxide concentration remains below the goethite formation threshold.

##### 3.3.1.1 Governing Equations

Hydroxide transport follows one-dimensional Fickian diffusion across the entire domain

$$\frac{\partial c\left(x,t\right)}{\partial t}\;=\;\frac{D_{eff}\partial^{2}c\left(x,t\right)}{\partial x^{2}},\;0<x<\infty$$ 1

where *c*(*x*,*t*) is the hydroxide concentration (mol m^–3^), *D*_eff_ is the effective hydroxide diffusivity in 1% agar gel (m^2^ s^–1^).

Three sharp reaction fronts separate distinct precipitation regions: magnetite at *x* = *d*_M_(*t*), green rust at *x* = *d*_GR_(*t*), and goethite at *x* = *d*_G_(*t*), with 0 < *d*_M_(*t*) < *d*_GR_(*t*) < *d*_G_(*t*).

Boundary conditions include the reservoir boundary *c*(0,*t*) =*c_b_* and moving boundary conditions:

$$\begin{array}{c} c(d_{M}(t),t)\;=\;c_{crit,M}\\ \begin{array}{c} c(d_{GR}(t),t)\;=\;c_{crit,GR}\\ c(d_{G}(t),t)\;=\;c_{crit,G} \end{array} \end{array}\;\;$$ 2

where *c*_crit,i_ is the hydroxide concentration required for sustained net precipitation (or sustained phase conversion) at that interface. These *c*_crit,*i*_ values should not be interpreted as universal thermodynamic constants (e.g., tabulated *K*_sp_values) because the onset of a solid phase in a gel depends on coupled hydrolysis/speciation, solubility, nucleation barriers, and activity effects at high ionic strength. Accordingly, we interpret *c*_crit,*i*_ as an effective interfacial (thermo-kinetic) threshold that aggregates “thermodynamic feasibility + kinetic accessibility” under the experimental conditions. The required hierarchy *c*_crit,G_ < *c*_crit,GR_ < *c*_crit,M_ is physically grounded in the well-known speciation contrast that Fe^3+^ hydrolyzes and precipitates at much lower pH than Fe^2+^, while mixed-valent phases and magnetite formation typically require more alkaline conditions.

Stefan conditions enforce mass balance at each front

3 $$-D_{eff}{\frac{\partial c}{\partial x}|}_{x=d_{i}^{-}}=\Lambda_{i}{ḋ}_{i},\left(i=G,GR,M\right)$$

Here, ${\overset{˙}{d}}_{i}$ represents the time derivative of *d*_*i*_, Λ_*i*_ represents the alkalinity demand (mol m^–3^), defined as the hydroxide consumption per unit volume of the gel matrix.

Initial conditions are *c*(*x*,0) = 0 and *d*_M_(0) = *d*_GR_(0) = *d*_G_(0) = 0.

##### 3.3.1.2 Similarity Solution

Introducing the similarity variable $\eta =\frac{x}{2\sqrt{D_{eff}t}}$ , the concentration profile is piecewise defined across four regions. For magnetite precipitation (0 ≤ *x* ≤ *d_M_* (*t*))

$$c_{I}(x,t)=c_{b}-(c_{b}-c_{crit,M})\frac{erf(\eta )}{erf(\lambda_{M})}$$ 4

For green rust precipitation (*d*_M_(*t*)≤ *x* ≤ *d*_GR_(*t*))

$$c_{II}\left(x,t\right)=c_{crit,M}-(c_{crit,M}-c_{crit,GR})\frac{\,erf(\eta )-erf{(\lambda }_{M})}{erf{(\lambda }_{GR})-erf{(\lambda }_{M})}\;$$ 5

For goethite precipitation (*d*_GR_(*t*)≤ *x* ≤ *d*_G_(*t*))

$$c_{III}\left(x,t\right)=c_{crit,GR}-(c_{crit,GR}-c_{crit,G})\frac{erf(\eta )-erf{(\lambda }_{GR})}{erf{(\lambda }_{G})-erf{(\lambda }_{GR})}\;$$ 6

Beyond the goethite front (*x* > *d*_G_(*t*)), *c*_IV_(*x*,*t*) = 0.

Each front advances parabolically

7 $$d_{i}\left(t\right)=2\lambda_{i}\sqrt{D_{eff}t},\;\left(i=G,GR,M\right)$$

The similarity parameters *λ*_*i*_ satisfy coupled transcendental equations. For the magnetite front

8 $$\frac{c_{b}-c_{crit,M}}{\Lambda_{M}}=\sqrt{\pi }\lambda_{M}\;\exp\left(\lambda_{M}^{2}\right)\;\mathrm{erf}\left(\lambda_{M}\right)$$

For the green rust front

9 $$\frac{c_{crit,M}-c_{crit,GR}}{\Lambda_{GR}}=\sqrt{\pi }\lambda_{GR}\;\exp\;\left(\lambda_{GR}^{2}\right)\;\left[\mathrm{erf}\left(\lambda_{GR}\right)-\mathrm{erf}\left(\lambda_{M}\right)\right]$$

For the goethite front

10 $$\frac{c_{crit,GR}-c_{crit,G}}{\Lambda_{G}}=\sqrt{\pi }\lambda_{G}\;\exp\left(\lambda_{G}^{2}\right)\;\left[\mathrm{erf}\left(\lambda_{G}\right)-\mathrm{erf}\left(\lambda_{GR}\right)\right]$$

These equations represent a flux balance at each moving boundary. The left-hand side is a dimensionless driving force (the hydroxide concentration drops across a front relative to its alkalinity demand), while the right-hand side is a function of the front velocity parameter (*λ*_*i*_). A larger driving force or a smaller Λ_*i*_ results in a larger *λ*_*i*_, corresponding to a faster-moving front.

##### 3.3.1.3 Front Velocities and Parameter Extraction

The front velocity prefactors are

11 $$\alpha_{i}\;=\;2\lambda_{i\;}\sqrt{D_{eff}}$$

Experimentally, front positions follow parabolic scaling

12 $$d_{i}\left(t\right)=\alpha_{i}t^{\beta_{i}}$$

with β_*i*_ ≈ 0.5 confirming diffusion-limited kinetics.

The alkalinity demands exhibit the hierarchy

13 $$\Lambda_{G}<\Lambda_{GR}\;<\Lambda_{M}$$

reflecting increasing hydroxide requirements from goethite to magnetite formation. Critical hydroxide concentrations follow

14 $$c_{crit,G}\;<\;{\;c}_{crit,GR}<c_{crit,M}<c_{b}$$

indicating that magnetite requires the highest hydroxide concentration for precipitation, while goethite forms at the lowest concentration. As discussed earlier, this hierarchy reflects the increasing thermodynamic stability of the reduced phases under higher alkalinity: goethite (α-FeOOH), a ferric oxyhydroxide, precipitates at the lowest [OH^–^], followed by the metastable mixed-valent green rust, with the most stable ferrimagnetic spinel, magnetite, forming only at the highest [OH^–^] closest to the reservoir.

In the weak-supply limit ((*c*_b_–*c*_crit,i_)/Λ_*i*_ ≪ 1), which corresponds to λ_*i*_ ≪ 1, the solution simplifies. Using the approximation $\sqrt{\pi }\lambda e^{\lambda^{2}}\mathrm{erf}\left(\lambda \right)\approx 2\lambda^{2}$ , the front prefactor becomes

$$\alpha_{i}\approx \sqrt{2\frac{\left(c_{b}-c_{crit,i}\right)D_{eff}}{\Lambda_{i}\,}}$$ 15

revealing the square-root dependence of front velocity on both hydroxide supply and alkalinity demand.

##### 3.3.1.4 Physical Interpretation

This theoretical framework (Scheme 2) establishes a quantitative bridge between macroscopic front dynamics and microscopic reaction parameters, providing a comprehensive physical interpretation of the sequential precipitation process. The model successfully explains the observed yellow → green → black region sequence as arising from successive precipitation thresholds, where each iron oxide phase nucleates at a characteristic critical hydroxide concentration. The front velocity hierarchy, with goethite advancing fastest (*α*_*G*_ > *α*_*GR*_ > *α*_*M*_), directly stems from the alkalinity demand hierarchy Λ_G_ < Λ_GR_ < Λ_*M*_, reflecting the increasing hydroxide consumption required for magnetite formation compared to goethite. This unequal consumption also accounts for the persistent widening of the goethite region, as the formation front outpaces the replacement front due to the lower hydroxide demand for goethite precipitation relative to its transformation to green rust.

Each moving boundary advances according to the **Stefan condition**: $-D_{eff}{\frac{\partial c}{\partial x}|}_{d_{i}^{-}}=\Lambda_{i}{ḋ}_{i}\left(t\right),\left\{i=G,GR,M\right\}$ , where Λ_*i*_ represents the phase-specific alkalinity demand (hydroxide consumption per unit gel volume) and ${ḋ}_{i}$ is the front velocity. The critical concentration hierarchy *c*_crit,G_ < *c*_crit,GR_ < *c*_crit,M_ reflects the increasing thermodynamic stability of iron oxide phases under higher alkalinity. The corresponding alkalinity demand hierarchy Λ_G_ < Λ_GR_ < Λ_M_ governs the front velocity sequence *α*_G_ > *α*_GR_ > *α*_M_, resulting in parabolic front advancement $d_{i}\left(t\right)\propto \sqrt{t}$ and progressive widening of the goethite region with time.

#### 3.3.2 Effect of Hydroxide Concentration

The theoretical framework established that the front velocity prefactor *α_i_* depends on the hydroxide supply *c*_*b*_, the effective diffusivity *D*_eff_, and the alkalinity demand Λ_*i*_ (eq 11). To test this dependence quantitatively, we systematically varied the outer hydroxide concentration *c*_*b*_ from 1.0 to 3.0 M while keeping the inner Fe loading constant ([Fe^2+^]:[Fe^3+^] = 0.10 M:0.20 M).

As predicted, increasing *c*_*b*_ enhanced the fitted prefactor *α*_*i*_ for all three fronts (Tables S4–S6), confirming that a greater hydroxide flux accelerates front propagation. The time exponents remained close to 0.5 for goethite and green rust (*β* ≈ 0.5), while a slightly higher value for magnetite (*β* ≈ 0.54) suggests a microstructure-driven acceleration, likely due to the formation of a porous magnetite precipitate that enhances local transport.

The Stefan model predicts a square-root dependence of *α*_*i*_ on hydroxide concentration for constant *D*_eff_ and Λ*_i_* (i.e., *α*_*i*_ ∝ *c*_*b*_^*m*^, *m* = 0.5) . To test this, we performed a log–log regression of ln*α_i_* versus ln*c_b_* across five base concentrations. The fitted scaling exponents were *m*_G_= 0.33 ± 0.05 *f* or goethite, *m*_GR_ = 0.34 ± 0.05 for green rust, and *m*_M_ = 0.36 ± 0.05 for magnetite (R^2^ ≈ 0.94).

These values are close to the theoretical prediction of 0.5, confirming that hydroxide flux is the primary driver of front velocity. The slightly sublinear scaling (*m* ≈ 0.33–0.36) likely reflects a mild decrease in the effective diffusivity, *D*_eff_, at higher ionic strengths due to increased gel tortuosity, which reduces hydroxide mobility. The close agreement between experiment and theory provides strong quantitative validation of the Stefan moving-boundary model.

Throughout this [NaOH] range, all regions maintained their sharp interfaces and characteristic color sequence (yellow → green → black), confirming that variations in hydroxide concentration modulate the front kinetics without altering the fundamental phase transformation pathway. The extracted alkalinity demands Λ_G_ ≈ 8000, Λ_GR_ ≈ 10000, Λ_M_ ≈ 15000 mol m^–3^ are physically realistic when considering the solid densities and hydroxide stoichiometries of the respective phases and represent the effective hydroxide consumption per unit volume of the gel matrix.

The magnitude of these Λ_i_ values is rationalized by considering that they represent the effective hydroxide consumption normalized to the total volume of the gel matrix, which is predominantly water (∼99 wt % for a 1.0 wt % agar gel). The actual solid precipitate occupies only a small fraction of this volume, yet the alkalinity demand accounts for the total OH^–^ required to transform the dissolved iron salts within a given gel volume into the dense, precipitated phase. This framework inherently incorporates the gel’s porous structure, where the local precipitate density is high, and the consumption is integrated over the macroscopic gel volume, which encompasses both the aqueous phase and the polymer network. Consequently, the extracted Λ_i_ values are substantially higher than the stoichiometric OH^–^ demand per unit volume of the pure solid phase alone, providing a consistent and physically meaningful parameter within the moving-boundary model.

#### 3.3.3 Effect of Iron Concentration

Having validated the model’s dependence on hydroxide supply, we next investigated the role of the reactive sink strength by varying the inner Fe concentration and the [Fe^2+^]/[Fe^3+^] ratio at a fixed outer electrolyte concentration ([NaOH] = 3.0 M).

The parabolic law *d*_*i*_ = *α*_*i*_ *t* ^*β_i_*^ held for all conditions, with *β*_*i*_ ≈ 0.5 confirming that the kinetics remained diffusion-limited (Tables S1–S3). As predicted by the Stefan model (*α*_i_∝Λ_*i*_^-1/2^), the front prefactor *α*_*i*_ decreased systematically with increasing total iron loading. This is because a higher concentration of iron cations increases the local alkalinity demand Λ*_i_*, slowing the advance of the reaction fronts for a given hydroxide flux. The extracted alkalinity demands consistently obeyed the hierarchy Λ_G_ < Λ_GR_ < Λ_M_, confirming that magnetite formation requires the greatest hydroxide consumption per transformed gel volume.

Beyond the front velocities, the [Fe^2+^]/[Fe^3+^] ratio had a profound impact on precipitation morphology. Fe^3+^-rich compositions (⩽1:2) produced a narrow goethite front, while near-equimolar mixtures (1.5:1.5 to 2:1) stabilized a distinct intermediate green rust region. As the system became enriched in Fe^2+^(≥2:1), the magnetite front thickened and darkened, signaling a higher hydroxide consumption that shortened the diffusion length.

Under highly Fe^2+^-rich conditions (≥2.5:0.5), the system transitions from a steady moving-boundary regime to an oscillatory one, producing periodic Liesegang bands within the magnetite region (Figure 3f–j). This occurs when the strong local hydroxide demand and slow diffusive replenishment create cycles of supersaturation, nucleation, and depletion at the advancing interface.

**Figure 3:**
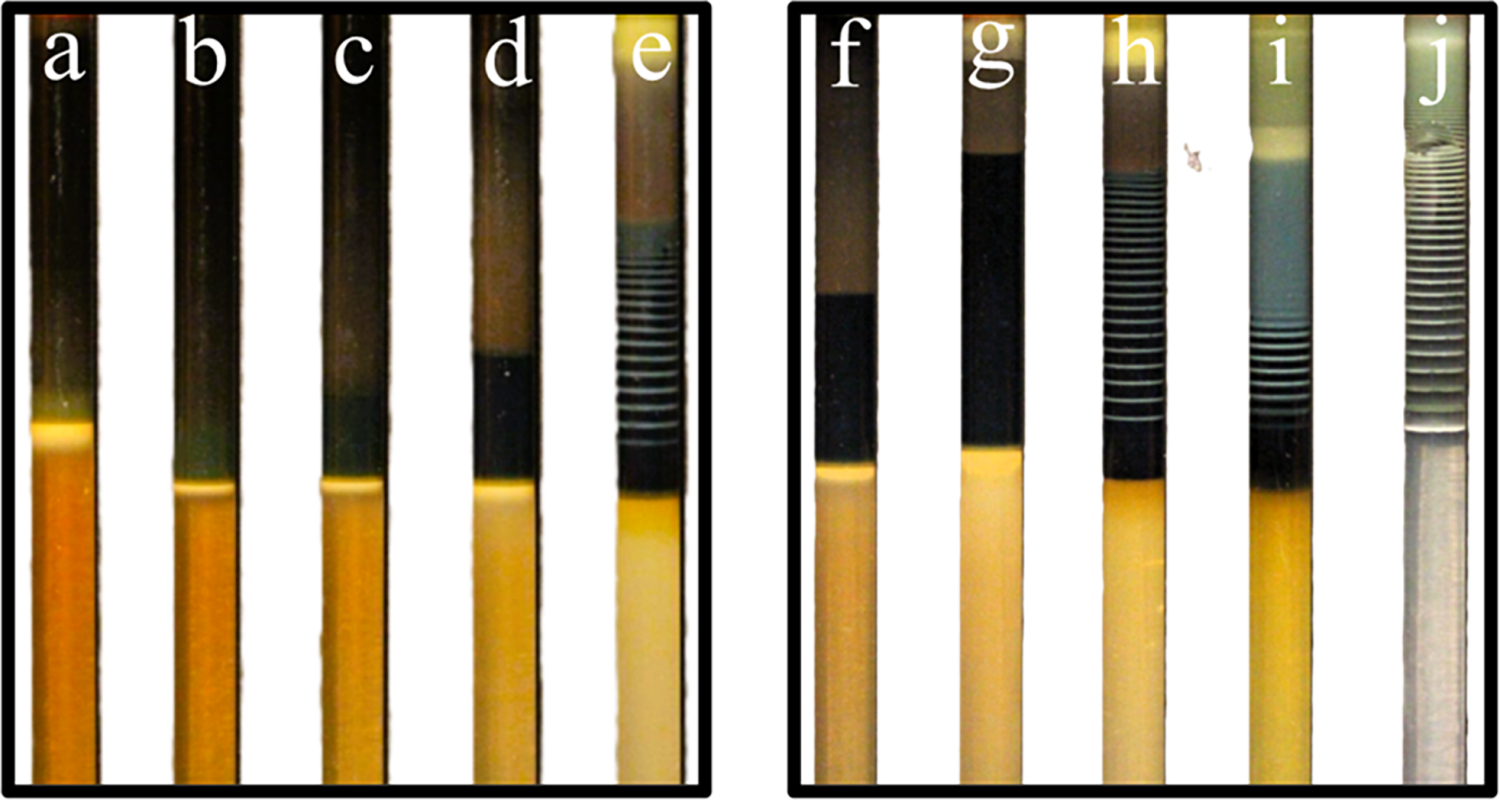
Precipitation–diffusion fronts in 1 wt % agar hydrogels at a fixed outer electrolyte concentration of [NaOH] = 3.0 M and varying Fe^2+^/Fe^3+^ ratios in the inner electrolyte. Panels (a–e) (left): 0.5:2.5, 1:2, 1.5:1.5, 2:1, and 2.5:0.5. Increasing Fe^2+^ content progressively shifts the phase sequence from goethite-rich (yellow) to green rust and magnetite-dominated (green → black) fronts. Fe^3+^-rich compositions favor goethite, whereas condition (e) exhibits the onset of periodic Liesegang banding, reflecting oscillatory supersaturation and nucleation within the advancing reaction front. Panels (f–j) (right): 2:1, 2.25:0.75, 2.5:0.5, 2.75:0.25, and 3:0. Further enrichment in Fe^2+^ enhances magnetite formation: (f–g) display black magnetite-dominated zones, (h) shows well-defined periodic Liesegang bands within the magnetite region, (i) yields mixed green rust/magnetite interfaces, and (j) presents highly regular, extensive Liesegang banding characteristic of oscillatory precipitation in diffusion-controlled systems.

This transition from steady moving boundaries to periodic Liesegang banding is captured dynamically in Movie S3. The video demonstrates that as the Fe^2+^ content increases (moving from left to right across the samples), the continuous magnetite front destabilizes into discrete, periodic discs. This behavior can be qualitatively understood within the context of Ostwald’s supersaturation (prenucleation) model. Under Fe^2+^-rich conditions, the precipitation of magnetite, which has the highest alkalinity demand (Λ_M_), rapidly depletes the local hydroxide concentration. When the rate of consumption exceeds the diffusive supply, the local supersaturation drops below the nucleation threshold, causing the front to halt (a “gap”). Precipitation only resumes downstream once diffusion replenishes the hydroxide levels to the critical supersaturation point (*S*_crit_).

Movie S3 thus serves as direct visual evidence of the competition between reaction consumption and diffusive supply. While a comprehensive theoretical modeling of these spacing laws is the subject of our ongoing work, the current observations confirm that the Fe^2+^ stoichiometry acts as the control parameter switching the system between steady (Stefan) and oscillatory (Liesegang) regimes.

The mean position of the precipitation zone still follows the *d* ∝ *t* ^1/2^ scaling of the Stefan model. However, each discrete band corresponds to a nucleation event that occurs when the local hydroxide concentration at the reaction plane recovers to the critical threshold *c*_crit_. Let *x*_*n*_ be the position of the *n*th band, formed at time *t*_*n*_. Applying the flux-balance condition with $ḋ\propto t^{-1/2}$ and integrating between successive events shows that the waiting times follow a geometric progression. Given the parabolic growth law *x* ∼ *t* ^1/2^, this temporal recurrence directly yields the classic Liesegang (Jablczynski) spacing law

16 $$x_{n}=Qx_{n-1}+\beta$$

Quantitative tracking of the pattern (Figure 3j) reveals a measured spacing coefficient of *Q* = 1.050 ± 0.004 (Figure S13). The corresponding spatial (Δ*x*_*n*_) and temporal (Δ*t*_*n*_) intervals increase with front age, explaining why bands become farther apart and less frequent as the front penetrates the gel. The obtained value for *Q* is very close to that measured for the ferrous hydroxide Liesegang system.^24^

In this framework, Liesegang banding is not a separate mechanism but a spatiotemporal modulation of the same diffusion-reaction process that governs the steady Stefan fronts. The transition is controlled by the [Fe^2+^]/[Fe^3+^] ratio: below a threshold (<2:1), the system maintains a steady front; above it (≥2.5:0.5), the interplay between high demand and limited supply drives the front into an oscillatory regime, producing the observed self-organized pattern.

#### 3.3.4 Effect of Gel Matrix

To further test the transport-limited nature of the process, we modulated the effective hydroxide diffusivity *D*_eff_ by varying the agar concentration from 0.5 to 2.0 wt %, while keeping the chemical composition constant. It is noteworthy that preliminary controls using gelatin and an agar-based LB Lennox formulation yielded the same qualitative goethite → green rust → magnetite spatial sequence, supporting that the cascade is not an artifact of agar-specific chemistry but reflects the imposed diffusion–reaction conditions.

As predicted by the Stefan model (*α_i_* ∝ *D*_eff_^1/2^), denser gels resulted in a systematic decrease of all front velocity prefactors α_*i*_. The transformation sequence remained unchanged, but the regions became sharper and narrower due to the reduced hydroxide flux. At 2.0 wt % agar, the effective diffusivity *D*_eff_ decreased by approximately 40% relative to the 0.5 wt % gel, which fully accounts for the observed slowdown of all fronts. Crucially, the extracted ratios of the alkalinity demands Λ_G_<Λ_G__R_<Λ_M_ remained constant across all gel densities.

This confirms that the agar matrix exclusively controls the transport kinetics without altering the underlying chemistry of the phase transformations. The gel acts as a tunable physical scaffold, where increased tortuosity at higher polymer content restricts ion mobility, thereby reducing *D*_eff_ and the overall front propagation rate, while leaving the intrinsic reaction demands Λ_*i*_unaffected.

#### 3.3.5 Goethite Region Widening Phenomenon

A distinctive feature of the precipitation sequence is the progressive thickening of the yellow goethite region with time. This macroscopic observation provides direct visual evidence of the kinetic hierarchy derived from our Stefan analysis.

The widening occurs because the rate of goethite formation at the leading front outpaces its rate of consumption at the trailing front. Within the moving-boundary framework, these two interfaces advance with different velocities. The leading boundary, where goethite precipitates from Fe^3+^ and OH^–^, is characterized by the alkalinity demand Λ_G_. The trailing boundary, where goethite is reductively dissolved and reprecipitated as green rust, is governed by the larger alkalinity demand Λ_G_ < Λ_GR_, as this transformation requires additional OH^–^ and the incorporation of Fe^2+^ diffusing from behind.

This difference in alkalinity demands results in different velocity prefactors, (*α*_lead_ > *α*_trail_). Consequently, the width of the goethite region, defined as the distance between these two boundaries, follows a parabolic growth law

$$w_{G}(t)=d_{G}(t)-d_{GR}(t)\approx (\alpha_{G}-\alpha_{GR})t^{1/2}$$ 17

The persistent and widening yellow zone is therefore a direct macroscopic manifestation of the alkalinity demand hierarchy Λ_G_ < Λ_GR_.

### 3.4 Transformation Mechanism and Pathway

SEM analysis (Figure 4A–C) corroborates the kinetic interpretation: goethite forms acicular crystallites aligned with the diffusion direction; green rust appears as hexagonal platelets; and magnetite emerges as discrete nanospheroids within a more porous matrix. The morphological evolution (needle → plate → sphere) illustrates a dissolution–reprecipitation mechanism rather than solid-state conversion, consistent with the quasi–stationary interfaces assumed in the Stefan model. The modest deviations of β from 0.5, thus encoding microstructural feedback: densification (goethite, green rust) slightly slows the front, whereas porosity generation (magnetite) slightly accelerates it.

**Figure 4:**
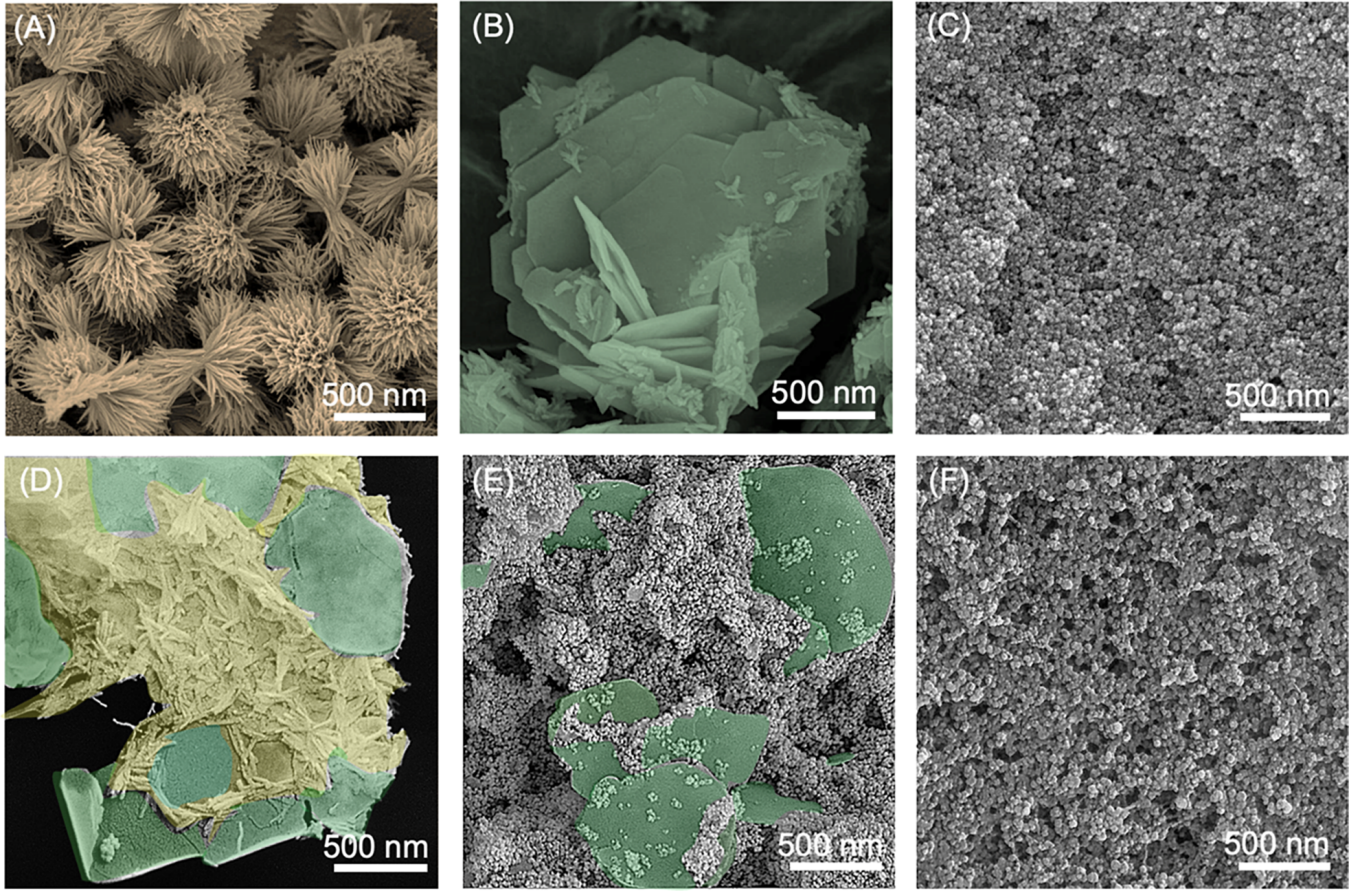
Scanning electron microscopy (SEM) images of iron oxides obtained from the precipitation–diffusion system under initial conditions of [Fe^2+^] = 0.10 M, [Fe^3+^] = 0.20 M, and [NaOH] = 3.0 M: (A) goethite (α-FeOOH), showing acicular crystals radiating into sheaf-like aggregates; (B) green rust, exhibiting characteristic hexagonal platelet morphology of layered double hydroxides; and (C) magnetite (Fe_3_O_4_), consisting of spherical nanoparticles forming dense, aggregated clusters; (D–F) illustrating the morphological transformation. The images are presented in false color to visually correlate each region with its corresponding macroscopic phase.

The sequential appearance of yellow, green, and black regions in the precipitation–diffusion system reflects a cascade of phase transformations driven by hydroxide diffusion, iron redox balance, and the gel’s confinement. Structural analyses confirmed the assignments of the regions as goethite (α-FeOOH), green rust (Fe^2+^–Fe^3+^ layered hydroxide), and magnetite (Fe_3_O_4_). The mechanistic pathway can thus be constructed by mapping the observed macroscopic patterns and microscopic evidence onto the underlying chemical reactions. Furthermore, we use the notation → to represent a chemical reaction that occurs without any imposed constraints.

At the advancing front, OH^–^ first encounters Fe^3+^, which hydrolyzes and precipitates as Fe­(OH)_3_ (R1) and then, by dehydration, yields α-FeOOH (R2)^25^

R1 $${Fe}^{3+}\left(aq\right)+3{OH}^{-}\left(aq\right)\;\to \;Fe{\left(OH\right)}_{3}\left(aq\right)$$

R2 $$Fe{\left(OH\right)}_{3}\left(aq\right)\;\to \;\alpha ‐FeOOH\left(s\right)+H_{2}O$$

Behind the goethite zone, Fe^2+^ diffuses inward and adsorbs on α-FeOOH surfaces. This facilitates partial dissolution and reprecipitation as green rust (R3), a sulfate-intercalated LDH^26,27^

R3 $$2\alpha ‐FeOOH\left(s\right)+4{Fe}^{2+}\left(aq\right)+6{OH}^{-}\left(aq\right)+2H_{2}O+{\text{SO}}_{4}^{2-}\left(aq\right)\;\to \;{Fe}_{4}^{2+}{Fe}_{2}^{3+}{\left(OH\right)}_{12}{\text{SO}}_{4}\left(s\right)$$

The terminal black region arises from the irreversible transformation of GR into magnetite via deprotonation and structural reorganization (R4)^28,29^

R4 $${Fe}_{4}^{2+}{Fe}_{2}^{3+}{\left(OH\right)}_{12}{\text{SO}}_{4}\left(s\right)+2{OH}^{-}\left(aq\right)\;\to \;{Fe}^{2+}{Fe}_{2}^{3+}O_{4}\left(s\right)+3{Fe}^{2+}{\left(OH\right)}_{2}+{\text{SO}}_{4}^{2-}\left(aq\right)+4H_{2}O$$

The production of Fe­(OH)_2_ as a metastable intermediate provides a coherent explanation for the observed Liesegang banding pattern under Fe^2+^-rich conditions. The periodic nature of Liesegang bands arises from supersaturation cycles and discrete nucleation events, which would naturally favor the sequential formation of Fe­(OH)_2_ followed by its transformation into magnetite. The characteristic green color of Fe­(OH)_2_ suspensions aligns with the greenish hues observed within the banding pattern, suggesting its transient presence contributes to the optical signature of these periodic structures. This intermediate subsequently oxidizes to magnetite, with the continued transformation explaining the persistent hydroxide demand measured at the magnetite front.

This entire transformation is governed by one-dimensional diffusion, which restricts the diffusion of hydroxide ions. Consequently, after a diffusion of 10 days, the total amount of OH^–^ ions transported is insufficient for the formation and growth of goethite. Additionally, the sulfate ion seems to have a structure-building impact in these acidic conditions; in its presence, the degree of hydrolysis is minimal. The extent of the effect depends on both the pH and the concentration of SO_4_^2–^.^4^

The hydrolysis of Fe^3+^ at a very low pH (≲3), a high concentration of the sulfate ion, and with monovalent cations, in this case NH_4_^+^, resulted in the creation of a Fe^III^-hydroxy-sulfate, also known as jarosite (R5)­

R5 $$3\alpha ‐FeOOH\left(s\right)+2{\text{SO}}_{4}^{2-}\left(aq\right)+3H^{+}\left(aq\right)+{NH}_{4}^{+}\left(aq\right)\;\to \;{NH}_{4}{Fe}_{3}{\left({\text{SO}}_{4}\right)}_{2}{\left(OH\right)}_{6}\left(s\right)+H_{2}O$$

Jarosite is an alunite group mineral with the chemical formula of ${MFe}_{3}{\left({\text{SO}}_{4}\right)}_{2}{\left(OH\right)}_{6}$ , which contains Fe^3+^ and NH_4_^+^ in the M site. Goethite and jarosite coexist for a brief period during the reaction process, but eventually, all the goethite is consumed, leaving only jarosite as the final product,^30^ as shown in (R5). This geochemically relevant pathway explains the eventual complete consumption of goethite in long-term experiments, with jarosite’s rhombohedral crystals (Figure S14) replacing goethite needles.

Beyond the synthetic pathway, this banded stabilization of green rust mimics the stratified redox interfaces found in natural soils and sediments, where Fe^2+^/Fe^3+^ cycling governs the fate of contaminants and the availability of nutrients. The controlled persistence of GR in confined spatial domains provides a laboratory model for studying electron transfer processes at environmental iron redox boundaries.

### 3.5 Microstructural Evolution and Dynamics

High-resolution SEM and CIF-based crystallographic analysis reveal that the sequential formation of goethite, green rust, and magnetite proceeds through a solution-mediated dissolution–reprecipitation mechanism. Each moving front corresponds to a specific coordination-to-coordination transformation governed by hydroxide diffusion, local redox exchange, and lattice reorganization (Scheme 3). The microscopic processes corroborate the Stefan moving-boundary picture developed earlier and quantitatively reproduce the observed hierarchical evolution of phases and morphologies (Scheme 2).

**Scheme 3:**
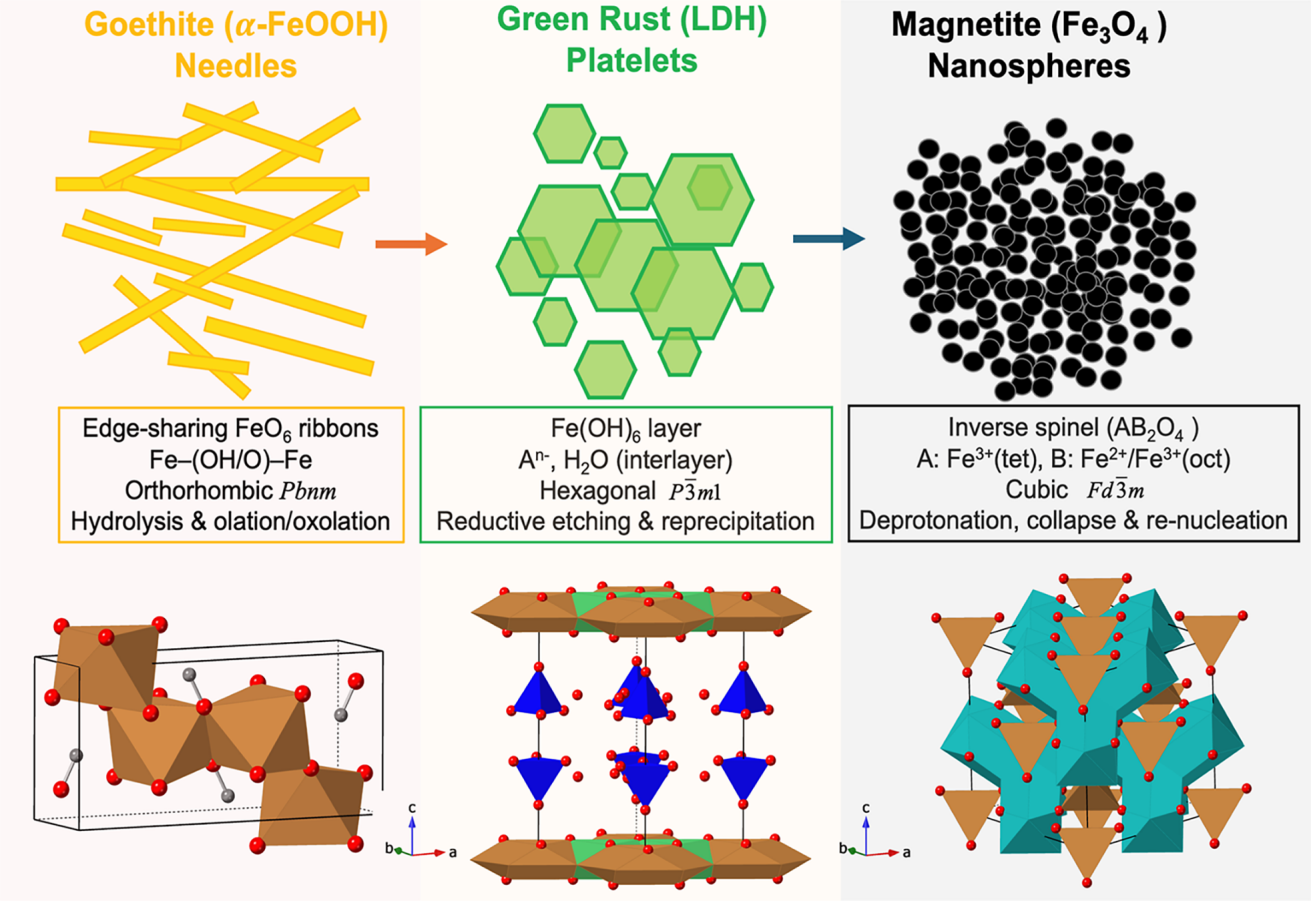
Crystallographic and Morphological Evolution Across Iron Oxide Transformation Fronts^a^

a Schematic illustration of the sequential structural reorganization during the goethite → green rust → magnetite transformation cascade. The progression reveals fundamental changes in coordination geometry: Edge-sharing FeO_6_ ribbons (goethite) → brucite-type Fe­(OH)_6_ layers (green rust) → inverse spinel framework (magnetite). Crystal symmetry: Orthorhombic (*Pbnm*) → hexagonal (*P*3̅*m1*) → cubic (*Fd*3̅*m*). Dimensionality: 1D needle-like chains → 2D layered sheets → 3D close-packed network. Transformation mechanisms: Hydrolysis/olation-oxolation → reductive dissolution-reprecipitation → deprotonation/collapse/re-nucleation. This hierarchical evolution from ferric oxyhydroxide through mixed-valent layered double hydroxide to ferrimagnetic spinel oxide directly corroborates the solution-mediated pathway inferred from the Stefan moving-boundary analysis

#### 3.5.1 Goethite Precipitation Front: Hydrolysis and Olation/Oxolation

At the leading yellow front, Fe^3+^ hydrolyzes and condenses under a steadily advancing hydroxide field to form α-FeOOH (goethite). The CIF of goethite (orthorhombic *Pbnm*) shows Fe^3+^ in distorted octahedral coordination (FeO_6_) with two distinct bond lengths: 1.93–1.98 Å (equatorial) and ≈2.09 Å (axial). These octahedra share edges to form double chains along [001], linked laterally by corner-sharing μ–OH bridges (Scheme 3).

This arrangement generates one-dimensional “ribbons” that polymerize via olation (Fe–OH–Fe) and oxolation (Fe–O–Fe) as hydroxide diffuses inward. The bonding transition follows (R6)­

$$Fe{\left(H_{2}O\right)}_{6}^{3+}\;\overset{{OH}^{-}}{\to }\;Fe\left(OH\right){\left(H_{2}O\right)}_{5}^{2+}\;\overset{condensation}{\to }\;Fe-\left(OH/O\right)-Fe$$ R6

leading to dehydration and network growth along [001].

The resulting anisotropy explains the needle- and spindle-like morphology observed by SEM (Figure 4A,B). Growth is controlled by the hydroxide flux (*J*_OH_– = −*D*_eff_∂*c*/∂*x*) and the interfacial consumption; the Stefan solution *d*_G_(*t*)∝*t* ^1/2^ captures the measured advance of the yellow region. Microscopically, goethite nucleates as dense bundles of oriented needles whose surfaces provide the template for subsequent reduction at the next interface.

#### 3.5.2 Goethite → Green Rust Transformation Front: Reductive Dissolution–Reprecipitation

At the green interface, inward-diffusing Fe^2+^ ions partially reduce Fe^3+^ within the goethite lattice, initiating reductive etching and reprecipitation of mixed-valent green rust (Fe^2+^–Fe^3+^ LDH) (Scheme 3). The green rust CIF (hexagonal *P*3̅*m1*) reveals brucite-type layers of edge-sharing Fe­(OH)_6_ octahedra with mixed Fe^2+^/Fe^3+^ occupancy (Fe^2+^/Fe^3+^ ≈ 2:1). Interlayer galleries contain anions (Cl^–^, SO_4_^2–^, or CO_3_^2–^) and water, with a basal spacing d_003_ ∼7.8 Å.

Mechanistically, Fe^2+^ attack breaks μ-OH and μ-O bridges in the goethite ribbons, releasing Fe­(OH)_x_ complexes that subsequently condense as LDH layers (R7)­

$$Fe\;-\;\left(OH/O\right)-{Fe}_{\left(ribbon\right)\;}\;\overset{{Fe}^{2+}/{OH}^{-}attack}{\to }Fe{\left(OH\right)}_{6}^{edge-shared}$$ R7

CIF analysis indicates in-plane Fe–Fe distances of ≈3.15 Å (edge-shared) and ≈4.5 Å across layers, confirming weaker interlayer cohesion and susceptibility to further dissolution.

Fe–O distances increase slightly to 2.05–2.12 Å (Fe^2+^) and 1.97–2.03 Å (Fe^3+^), yielding an average of ≈2.07 Å, consistent with partial reduction and hydration.

This transformation proceeds by dissolution–reprecipitation rather than solid-state conversion: SEM images (Figure 4D) show corroded goethite spindles surrounded by hexagonal LDH platelets, often nucleated epitaxially on the remnants of the needles (Scheme 3). Chemically, the interface operates under a higher alkalinity demand Λ_GR_ > Λ_G_, as both Fe^2+^ incorporation and OH^–^ coordination are required. Morphologically, the system evolves from 1D chains to 2D plates, mirroring the change in crystallographic symmetry (orthorhombic → hexagonal). The resulting platelets are thin (<100 nm) and laterally extended, stabilized by the layered structure and interlayer water.

#### 3.5.3 Green Rust → Magnetite Front: Deprotonation, Dehydration, and Spinel Reorganization

At the innermost black front, further OH^–^ influx and partial oxidation trigger the collapse of the LDH framework and the renucleation of magnetite (Fe_3_O_4_). The magnetite CIF (cubic *Fd*3̅*m*) shows a close-packed O^2–^ sublattice with Fe^3+^ occupying tetrahedral (A) sites (Fe–O ≈ 1.88 Å) and mixed Fe^2+^/Fe^3+^ in octahedral (B) sites (Fe–O ≈ 2.05 Å) (Scheme 3). This rearrangement corresponds to a fundamental change in bonding topology (R8)­

$$Fe{\left(OH\right)}_{6}^{layered}\;\overset{deprotonation/dehydration}{\to }\;{Fe}_{tet}^{3+}\left(O_{4}\right)+{Fe}_{oct}^{2+/3+}{\left(O\right)}_{6}$$ R8

During this transition, interlayer water and anions are expelled, μ-OH bonds are cleaved, and a 3D Fe–O framework forms. Morphologically, the layered platelets disintegrate into aggregates of magnetite nanospheres (10–30 nm), as observed in Figure 4E,F. The isotropic spinel symmetry eliminates directional growth, resulting in coalescence into dense, black aggregates.

Among the three stages, this interface exhibits the largest alkalinity demand (Λ_M_) and the highest effective reaction rate, consistent with the strong Fe^2+^ consumption and electron redistribution.

Together, these transformations establish a coherent hierarchy of chemical and structural complexity (Scheme 3): 1D edge-sharing chains → 2D brucite-like layers → 3D spinel network, accompanied by an increasing Fe^2+^/Fe^3+^ ratio and Fe–O distance modulation.

Under Fe^2+^-rich conditions, the final step enters an oscillatory regime where supersaturation cycles produce periodic Liesegang bands, governed by the same Stefan diffusion–reaction balance that controls the mean front motion.

The progressive bond breaking, redox equilibration, and renucleation across the three fronts thus provide a direct microscopic realization of the moving-boundary kinetics derived theoretically in this work.

## 4 Conclusion

Agar-gel precipitation–diffusion provides a quantitative, room-temperature framework for resolving the sequential formation and transformation of iron oxides. The macroscopic propagation of precipitation fronts obeys Stefan-type diffusion–reaction kinetics, with front prefactors scaling as $\alpha_{i}\propto {\left(D_{eff}c_{b}/\Lambda_{i}\right)}^{1/2}$ . The hierarchy of alkalinity demands Λ_G_ < Λ_GR_ < Λ_M_ rationalizes the observed sequence goethite → green rust → magnetite and explains the dependence of front velocity on hydroxide supply and iron loading. On the microscopic level, SEM imaging reveals a dissolution–reprecipitation pathway from goethite spindles to green rust platelets to magnetite nanospheres, directly corroborating the moving-boundary picture derived from the Stefan analysis.

Under Fe^2+^-rich conditions, the system enters a regime of periodic supersaturation, producing Liesegang bands whose spacing and regularity are quantitatively consistent with the diffusion–reaction balance of the Stefan framework. The geometric spacing law *x*_*n*+1_ = *px*_*n*_ with *p* ≈ 1.05 links the oscillatory precipitation directly to the underlying transport kinetics, demonstrating that pattern formation and interface motion arise from the same mechanistic origin.

Finally, we note that the transformation cascade reported here is not an artifact of the 1D reactor geometry. Preliminary validation experiments in a 2D radial diffusion cell have reproduced the identical spatial sequence (Yellow → Green → Black), confirming that the phase evolution is governed by the intrinsic reaction-diffusion coupling rather than dimensional constraints.

Building on these findings, future efforts will pursue two directions: (i) Liesegang physics: we will systematically map the banding regime by varying the [Fe^2+^]/[Fe^3+^] ratio, [NaOH], ionic strength, and gel tortuosity; quantify spacing ratios and induction times; and compare the results with linear-stability and supersaturation–relaxation models formulated within the Stefan framework. (ii) Doped ferrites (MFe_2_O_4_): we will extend the reaction–diffusion method to Co-, Ni-, Cu-, and Zn-doped ferrites, exploiting spatial confinement to tune crystallite size, cation distribution, and magnetic anisotropy. Magnetic characterization (VSM/SQUID) will focus on optimizing saturation magnetization and specific absorption rate (SAR) for magnetic hyperthermia applications, including hypothermic cell killing. Adjusting dopant level, gel porosity, and diffusion conditions will allow precise control over structure–property relationships.

In summary, this work integrates reaction–diffusion physics and phase-transformational chemistry into a coherent, quantitative framework, opening a pathway toward the controlled synthesis of functional ferrite nanomaterials with tunable spatial organization and magnetic performance.

## Supplementary Material









## Notes

Experimental setup for evaluation of Fe^2+^ stability, SEM images of the different inner iron concentrations, SEM images of the different outer hydroxide concentrations, SEM images of the various agar content, Diffusion propagations of the different base identity, SEM images of the iron oxide nanoparticles for the different bases, optical micrograph of the interface width between goethite and green regions, digital extraction and spacing law plot of the periodic Liesegang bands, XRD patterns before and after N_2_ sorption analysis, reflectance and Fourier transform of the Jarosite particles, fitting tables of the diffusion profiles at various inner iron and different outer hydroxide concentrations and textural and physicochemical properties of the three phases (PDF)
Effect of Outer Hydroxide Concentration (MP4)
Effect of Inner Iron Concentration (MOV)
Effect of Fe­(II)/Fe­(III) Ratio (Transition to Liesegang Banding) (MOV)

## Abbreviations

GR: green rust
RDF: reaction-diffusion framework
P–D: precipitation–diffusion
LDH: layered double hydroxide.
