# Supplementary material for: Transformation Cascades in Iron Oxides: Quantitative Resolution of Sequential Precipitation Using the Reaction-Diffusion Framework
Source: ACS Omega. 2026 Feb 5;11(6):10320–34. doi: 10.1021/acsomega.5c11472 (PMC12917817; doi:10.1021/acsomega.5c11472)
Supplement: Supplementary file 1 [file ao5c11472_si_001.pdf]

Transformation Cascades in Iron Oxides: Quantitative Resolution of Sequential Precipitation Using the Reaction-Diffusion Framework

Nour Abi Aad and Mazen Al-Ghoul[*]

Department of Chemistry, American University of Beirut, Beirut, Lebanon

Corresponding Author

[*] mazen.ghoul@ aub.edu.lb

SUPPORTING INFORMATION

SUPPORTING INFORMATION TABLE OF CONTENTS

FIGURES

- **Figure S1:** Evaluation of Fe^{2+} stability using a thiocyanate indicator.
- **Figure S2:** SEM images of goethite formation under varying inner iron concentrations.
- **Figure S3:** SEM images of green rust formation under varying inner iron concentrations.
- **Figure S4:** SEM images of magnetite formation under varying inner iron concentrations.
- **Figure S5:** SEM images of goethite formation under varying outer hydroxide concentrations.
- **Figure S6:** SEM images of green rust formation under varying outer hydroxide concentrations.
- **Figure S7:** SEM images of magnetite formation under varying outer hydroxide concentrations.
- **Figure S8**: SEM images of magnetite nanoparticles and front images under varying agar gel content (0.5–2.0 wt%).
- **Figure S9**: Temporal evolution of diffusion fronts using different alkaline sources (NaOH, KOH, Ammonia).
- **Figure S10:** SEM images of iron oxides formed using Ammonia and KOH.
- **Figure S11:** PXRD patterns of goethite and magnetite before and after N_2 sorption activation.
- **Figure S12:** Optical micrograph quantifying the interface width between goethite and green rust regions.
- **Figure S13**: Digital extraction of periodic bands and the Jablczynski spacing law plot for the Liesegang instability.
- **Figure S14.** Characterization of Jarosite (SEM, PXRD, ATR-FTIR, and UV-Vis) formed after long-term diffusion.

TABLES

- **Table S1:** Diffusion profile fitting parameters (α, β) for Goethite at different inner iron concentrations.
- **Table S2:** Diffusion profile fitting parameters (α, β) for Green Rust at different inner iron concentrations.
- **Table S3:** Diffusion profile fitting parameters (α, β) for Magnetite at different inner iron concentrations.
- **Table S4:** Diffusion profile fitting parameters (α, β) for Goethite under varying outer hydroxide concentrations.
- **Table S5:** Diffusion profile fitting parameters (α, β) for Green Rust under varying outer hydroxide concentrations.

- **Table S6:** Diffusion profile fitting parameters (α, β) for Magnetite under varying outer hydroxide concentrations.
- **Table S7:** Summary of textural and physicochemical properties (Surface Area, Pore Volume, Pore Diameter) for the three phases.

VIDEO CAPTIONS

- **Video S1:** Effect of Outer Hydroxide Concentration.
- **Video S2:** Effect of Inner Iron Concentration.
- **Video S3:** Effect of Fe(II)/Fe(III) Ratio (Transition to Liesegang Banding).

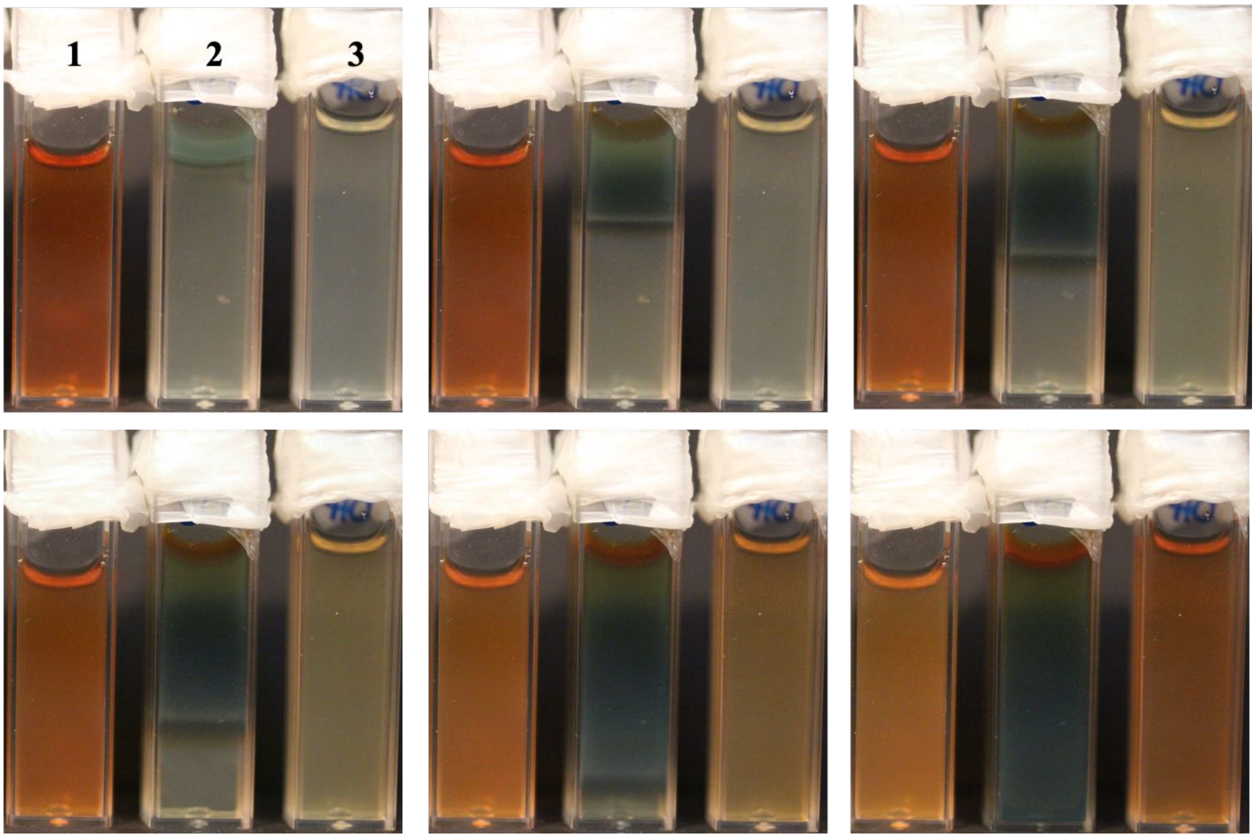

Figure S1. Evaluation of Fe^{2+} stability using a thiocyanate indicator. **(1)** Positive control (Fe^{3+} + SCN^-) showing the characteristic red complex. **(2)** The experimental system (Fe^{2+} + gel + SCN^- + diffusing NaOH), showing that the bulk gel remains free of the red oxidation product, protected by the interfacial precipitate. **(3)** Negative control (Fe^{2+} + SCN^- exposed to air) showing gradual oxidation throughout the gel in the absence of the reaction front.

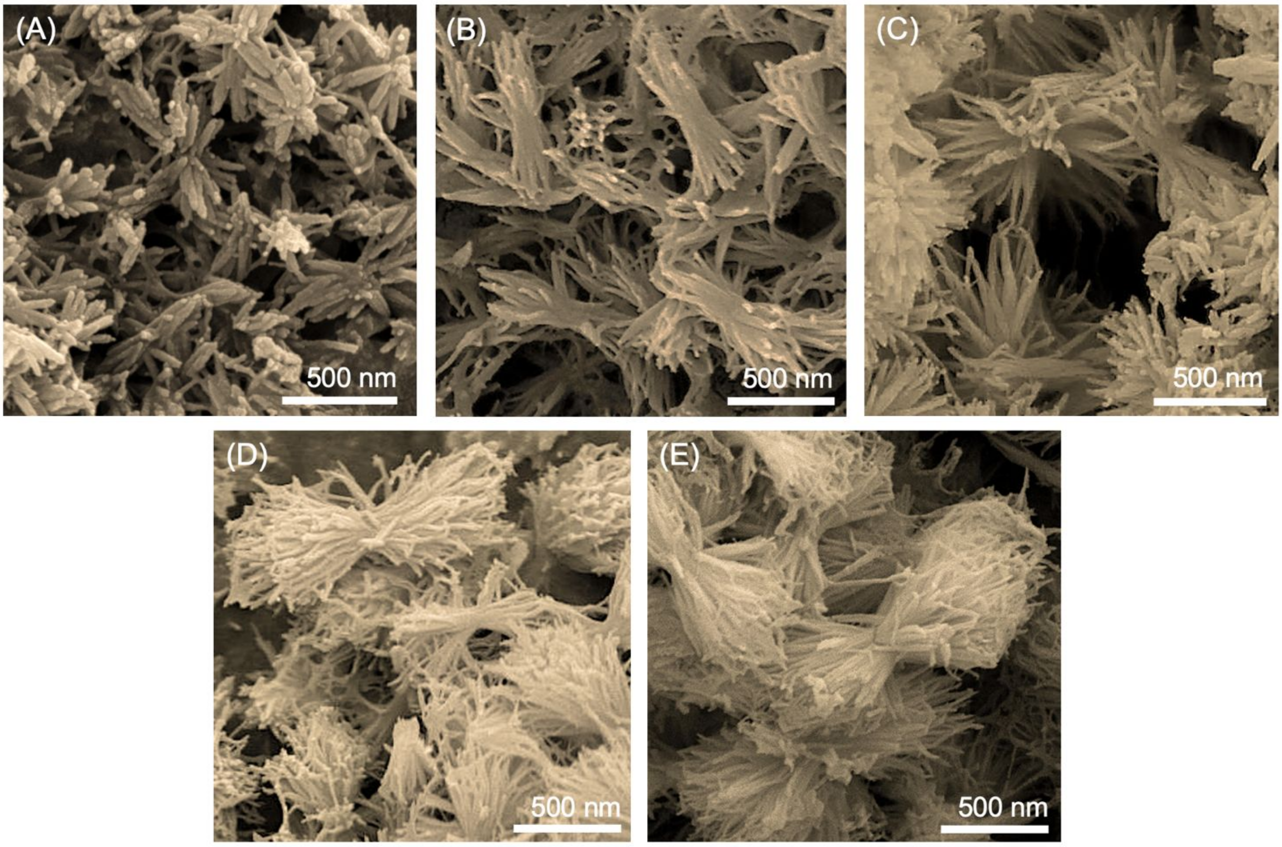

Figure S2. Scanning electron microscopy (SEM) images of fixed outer electrolyte concentration of 3.0 M NaOH and varying inner concentrations of Fe^{2+}/Fe^{3+} salts for goethite with (a) $[Fe^{2+}]$ = 0.04 M, $[Fe^{3+}]$ = 0.08 M; (b) $[Fe^{2+}]$ = 0.07 M, $[Fe^{3+}]$ = 0.14 M; (c) $[Fe^{2+}]$ = 0.10 M, $[Fe^{3+}]$ = 0.20 M; (d) $[Fe^{2+}]$ = 0.15 M, $[Fe^{3+}]$ = 0.30 M; and (e) $[Fe^{2+}]$ = 0.20 M, $[Fe^{3+}]$ = 0.40 M. The images are presented in false color to visually correlate each region with its corresponding macroscopic phase.

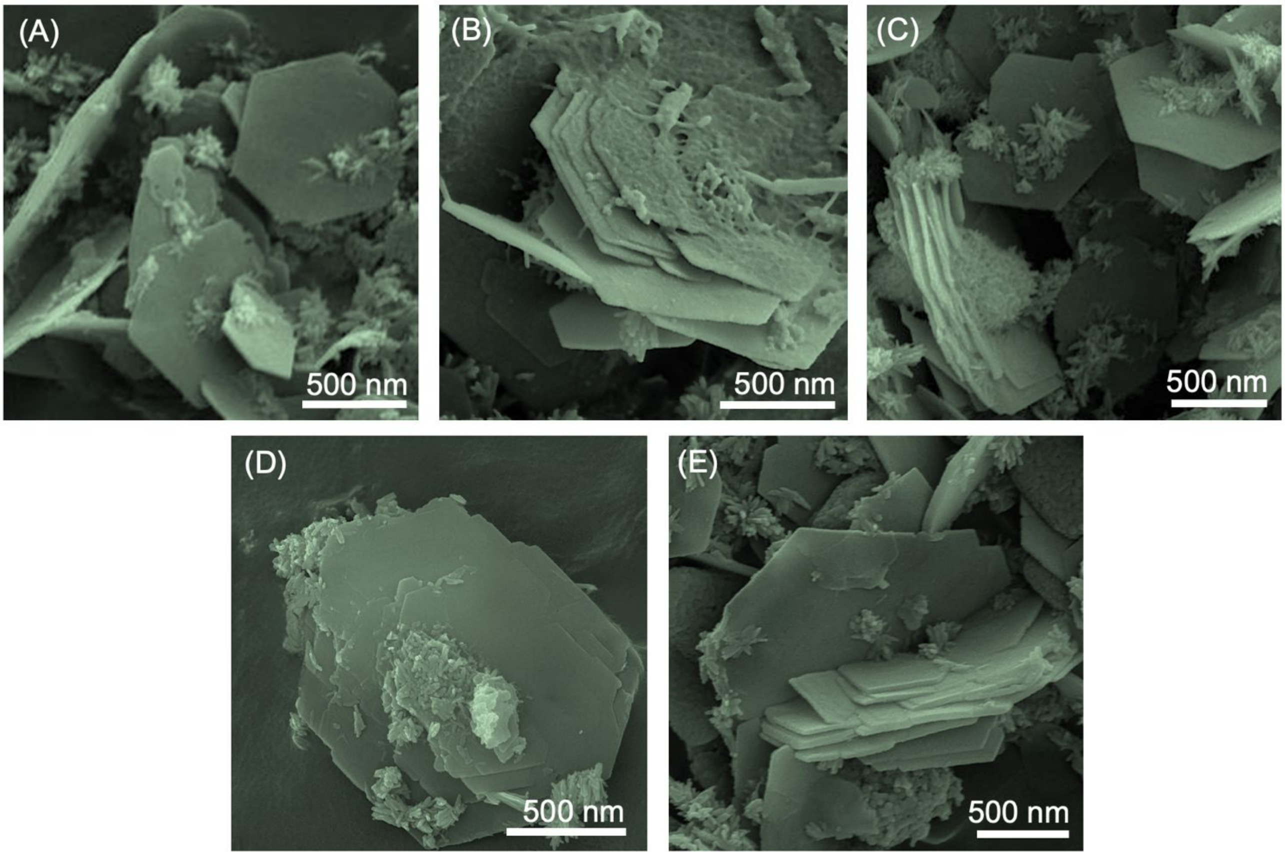

Figure S3. Scanning electron microscopy (SEM) images of fixed outer electrolyte concentration of 3.0 M NaOH and varying inner concentrations of Fe^{2+}/Fe^{3+} salts for green rust with (a) [Fe^{2+}] = 0.04 M, [Fe^{3+}] = 0.08 M; (b) [Fe^{2+}] = 0.07 M, [Fe^{3+}] = 0.14 M; (c) [Fe^{2+}] = 0.10 M, [Fe^{3+}] = 0.20 M; (d) [Fe^{2+}] = 0.15 M, [Fe^{3+}] = 0.30 M; and (e) [Fe^{2+}] = 0.20 M, [Fe^{3+}] = 0.40 M. The images are presented in false color to visually correlate each region with its corresponding macroscopic phase.

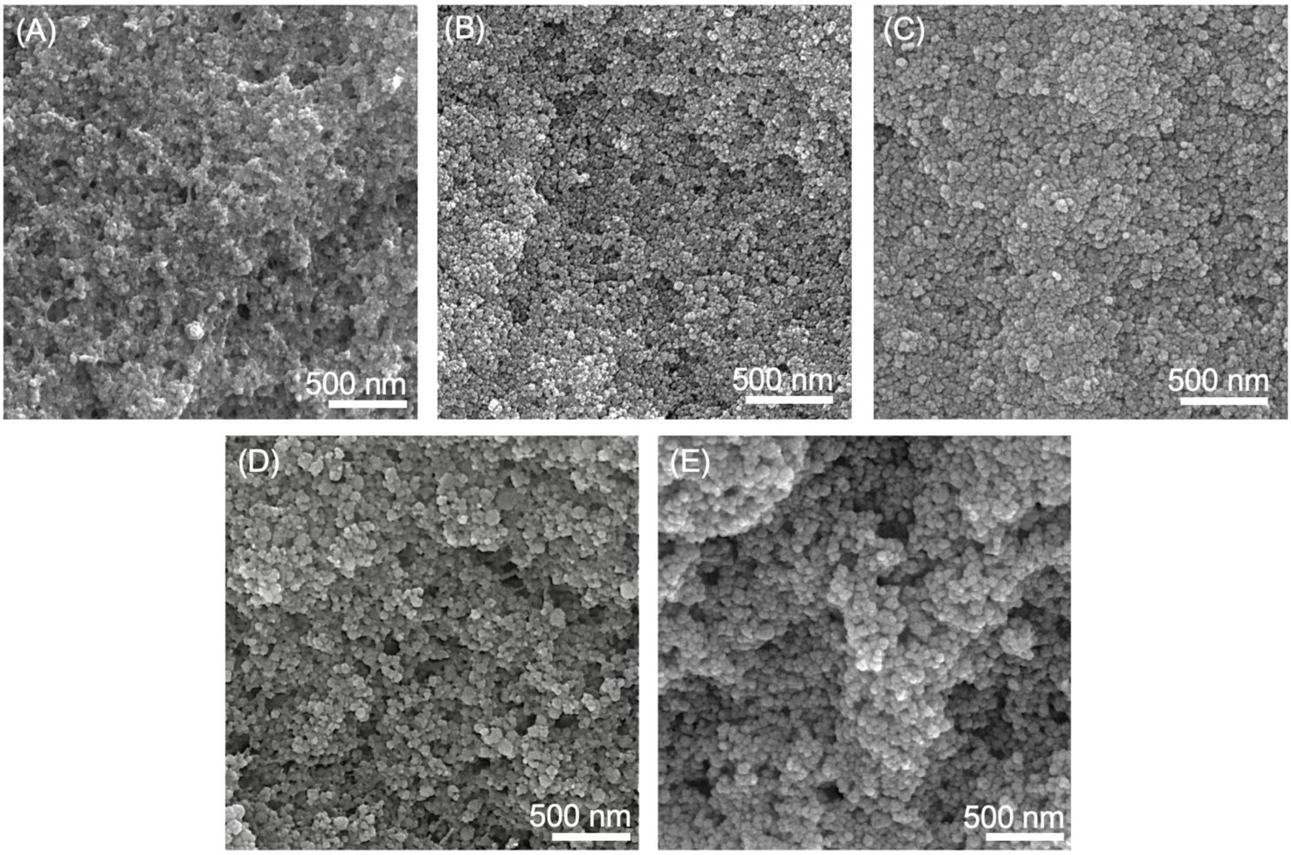

Figure S4. Scanning electron microscopy (SEM) images of fixed outer electrolyte concentration of 3.0 M NaOH and varying inner concentrations of Fe^{2+}/Fe^{3+} salts for magnetite with (a) $[Fe^{2+}]$ = 0.04 M, $[Fe^{3+}]$ = 0.08 M; (b) $[Fe^{2+}]$ = 0.07 M, $[Fe^{3+}]$ = 0.14 M; (c) $[Fe^{2+}]$ = 0.10 M, $[Fe^{3+}]$ = 0.20 M; (d) $[Fe^{2+}]$ = 0.15 M, $[Fe^{3+}]$ = 0.30 M; and (e) $[Fe^{2+}]$ = 0.20 M, $[Fe^{3+}]$ = 0.40 M.

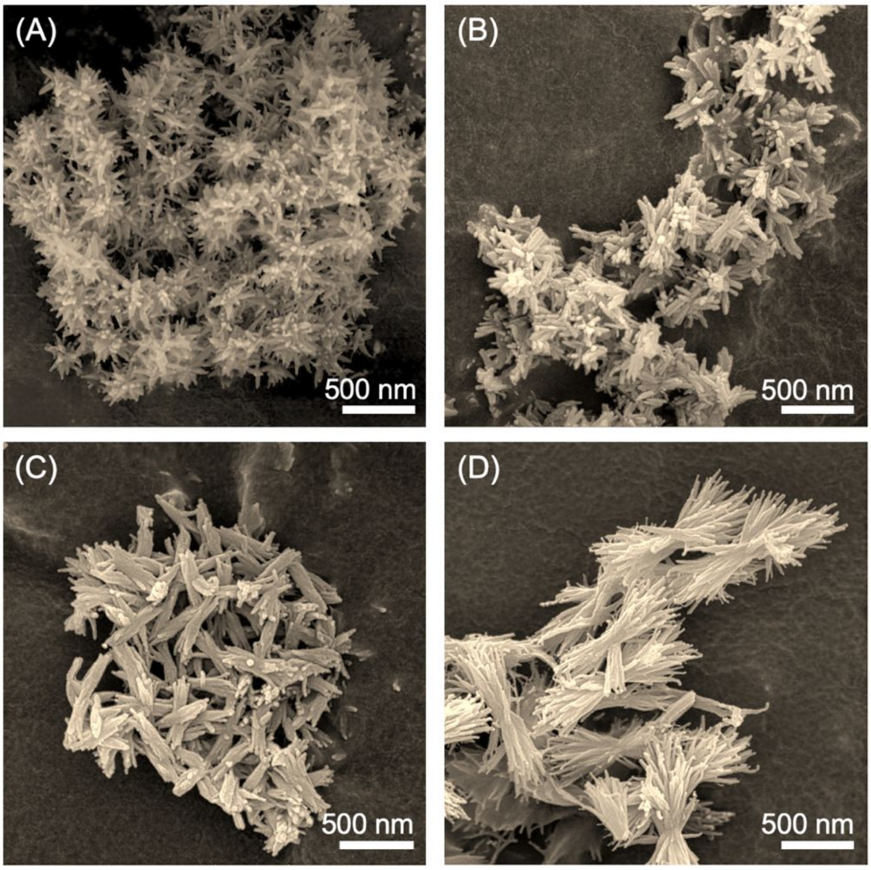

Figure S5. Scanning electron microscopy (SEM) images of 1.0 wt% agar hydrogels containing a fixed inner electrolyte concentration of [Fe^{2+}] = 0.10 M and [Fe^{3+}] = 0.20 M, with varying outer electrolyte concentrations of NaOH for goethite: (A) 1.0 M, (B) 1.5, (C) 2.0 M, (D) 2.5 M. The images are presented in false color to visually correlate each region with its corresponding macroscopic phase.

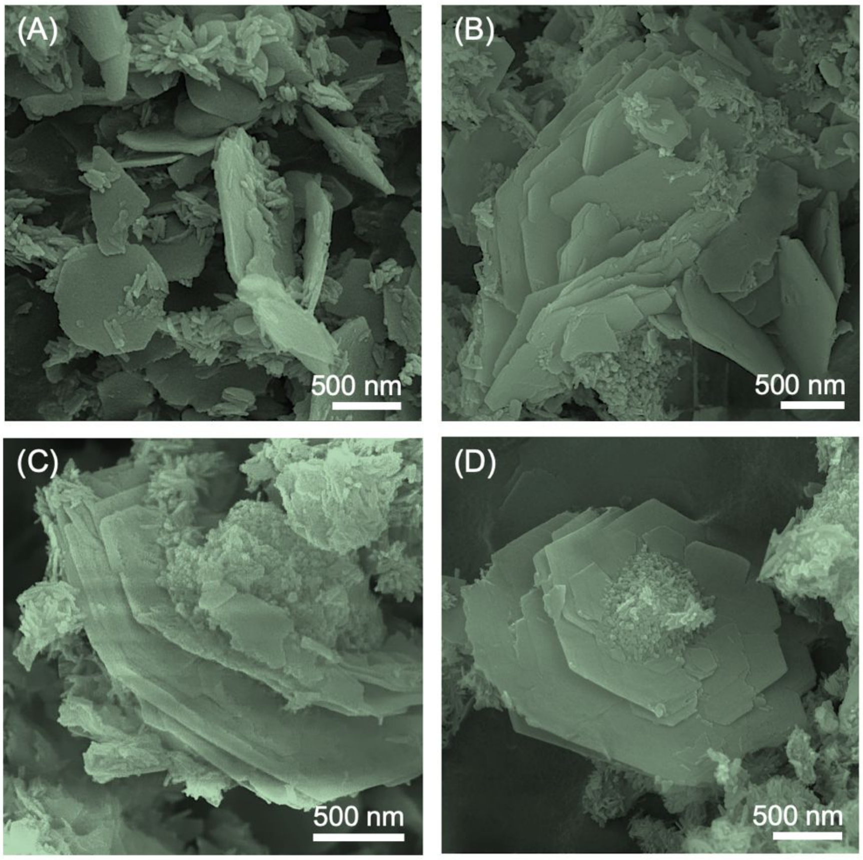

Figure S6. Scanning electron microscopy (SEM) images of 1.0 wt% agar hydrogels containing a fixed inner electrolyte concentration of [Fe^{2+}] = 0.10 M and [Fe^{3+}] = 0.20 M, with varying outer electrolyte concentrations of NaOH for green rust: (A) 1.0 M, (B) 1.5 M, (C) 2.0 M, (D) 2.5 M. The images are presented in false color to visually correlate each region with its corresponding macroscopic phase.

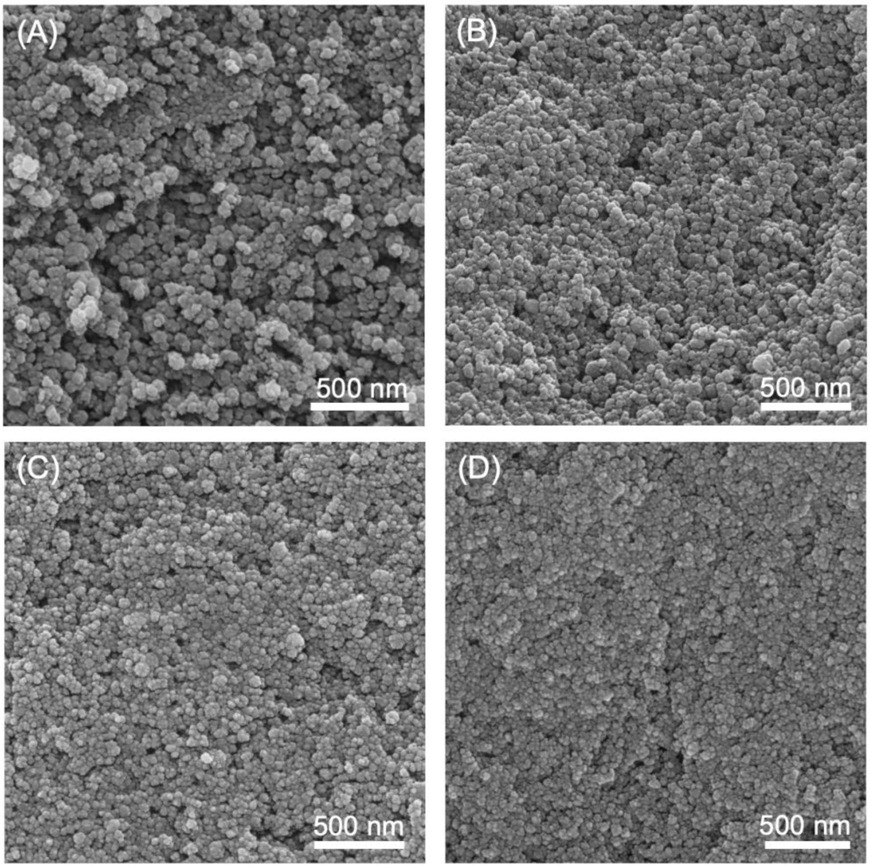

Figure S7. Scanning electron microscopy (SEM) images of 1.0 wt% agar hydrogels containing a fixed inner electrolyte concentration of [Fe^{2+}] = 0.10 M and [Fe^{3+}] = 0.20 M, with varying outer electrolyte concentrations of NaOH for magnetite: (A) 1.0 M, (B) 1.5 M, (C) 2.0 M, (D) 2.5 M.

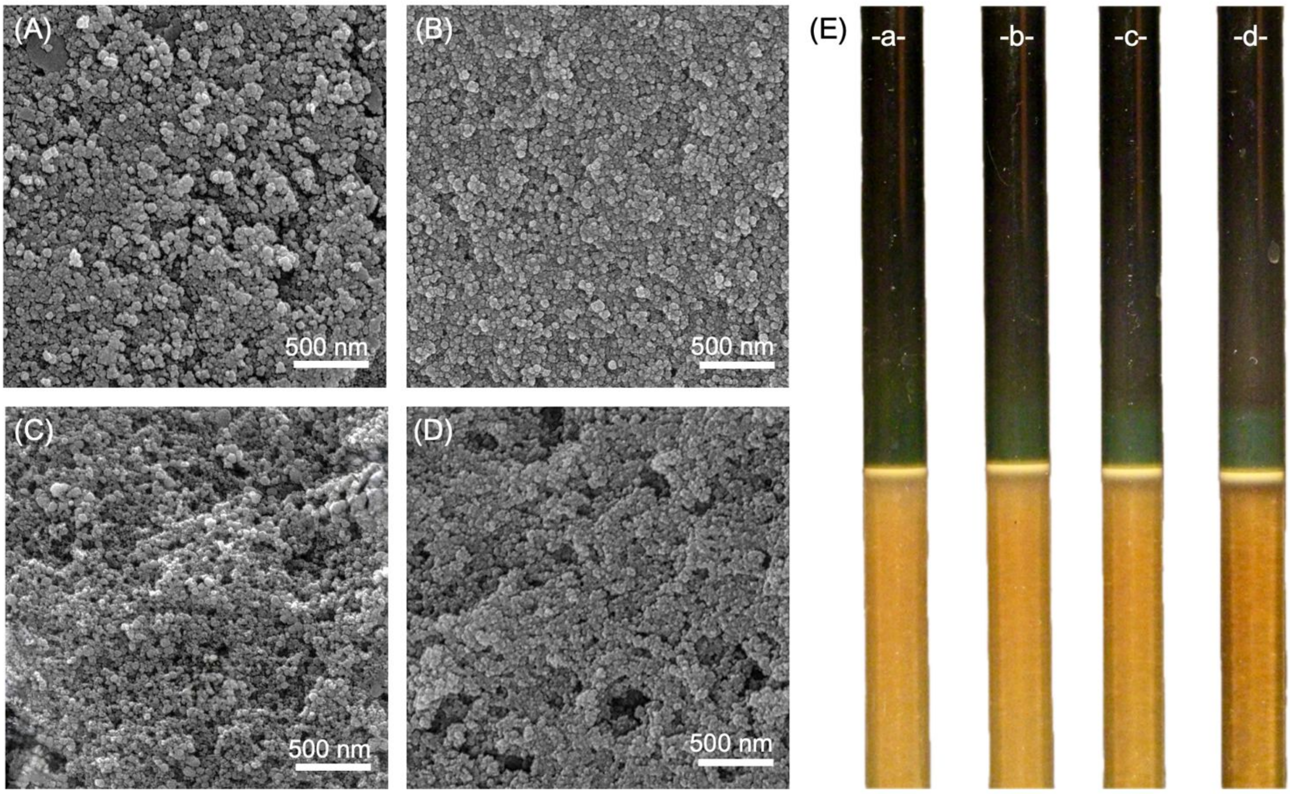

Figure S8. Scanning electron microscopy (SEM) images of various agar content: (A) 0.5 wt%, (B) 1.0 wt%, (C) 1.5 wt%, and (D) 2.0 wt%. The aggregated magnetite nanoparticles show a minor but clear trend of increasing particle size with increasing agar concentration. (E) Precipitation–diffusion fronts in agar hydrogels containing a fixed inner electrolyte concentration of [Fe^{2+}] = 0.10 M and [Fe^{3+}] = 0.20 M, with a fixed outer electrolyte concentration of [NaOH] = 3.0 M, with varying agar content from (A) to (D), respectively.

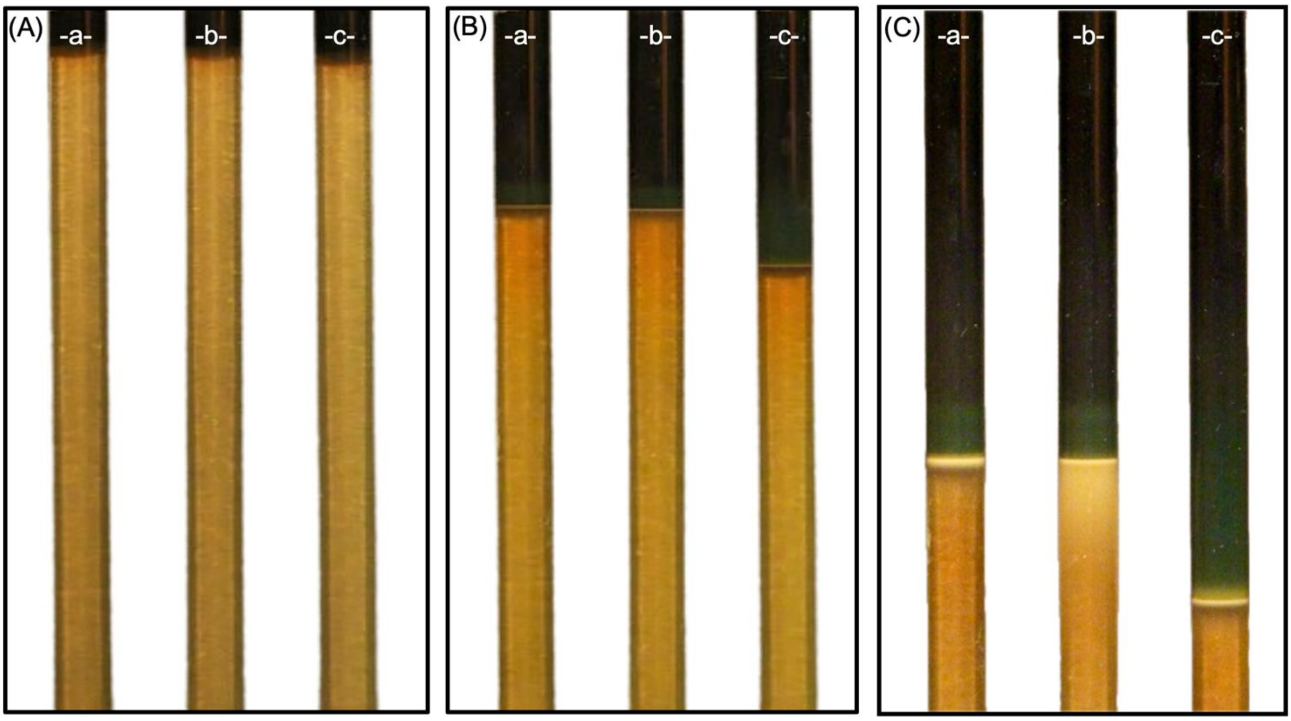

Figure S9. Precipitation–diffusion fronts in 1.0 wt% agar hydrogels containing a fixed inner electrolyte concentration of [Fe^{2+}] = 0.10 M and [Fe^{3+}] = 0.20 M, using different outer bases: (a) NaOH, 3.0 M; (b) KOH, 3.0 M; (c) ammonia (NH_3, 25%), ~13 M. The temporal evolution of the diffusion fronts for the different bases at (A) 1 hour, (B) 24 hours, and (C) 96 hours. The sequential color bands, yellow (goethite), green (green rust), and black (magnetite), reflect the phase transformations along the advancing reaction–diffusion front. The ammonia front shows a faster diffusion than KOH and NaOH, highlighting the effect of the OH^- supply on the system.

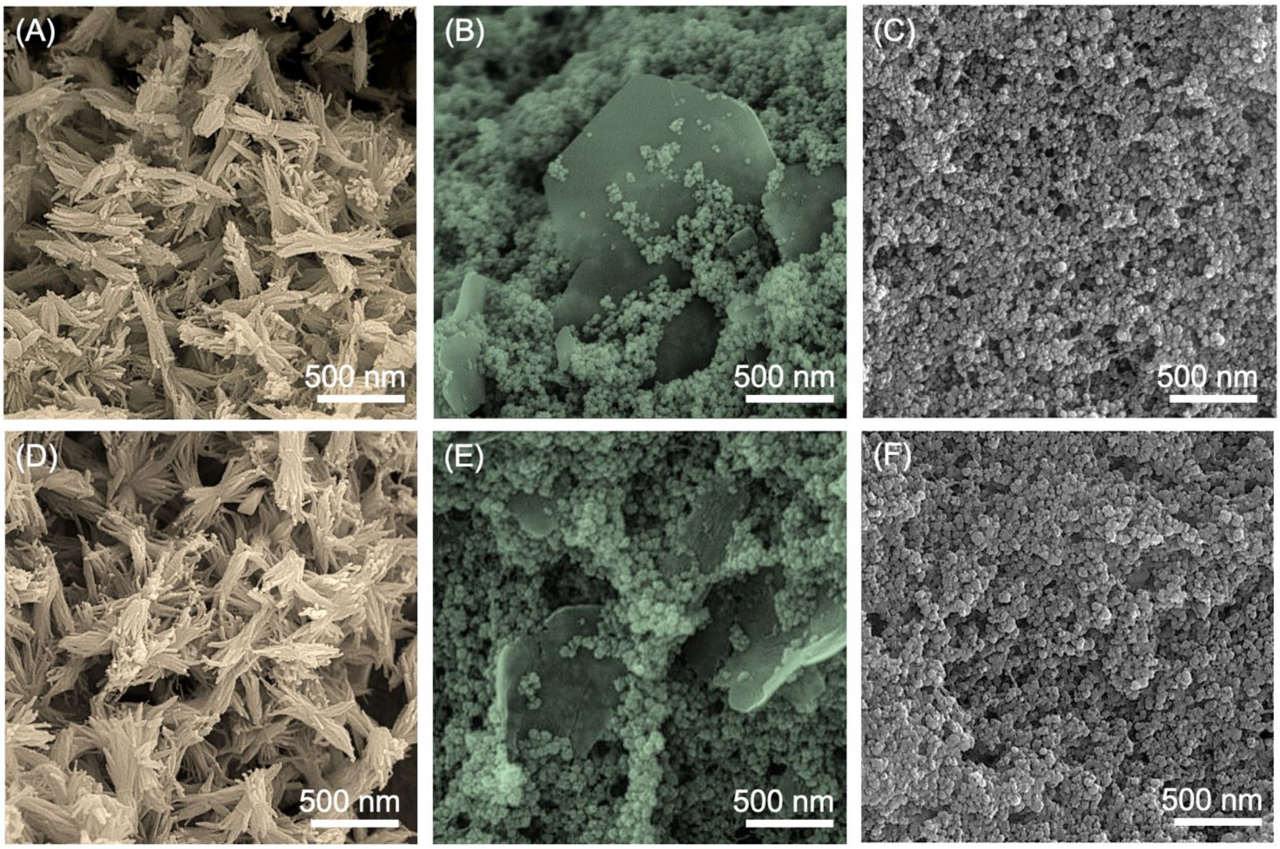

Figure S10. Scanning Electron microscope (SEM) images of iron oxides obtained under initial conditions [Fe^{2+}]= 0.10 M, [Fe^{3+}] = 0.20 M, with (A-C) [NH_3] ~13 M and (D-F) [KOH] = 3.0 M. The goethite (α-FeOOH) shows crystal needles with (B and E) the dissolution of the hexagonal sheets into (C) and (F) spherical aggregated magnetite nanoparticles, capturing the transition zone. The images are presented in false color to visually correlate each region with its corresponding macroscopic phase.

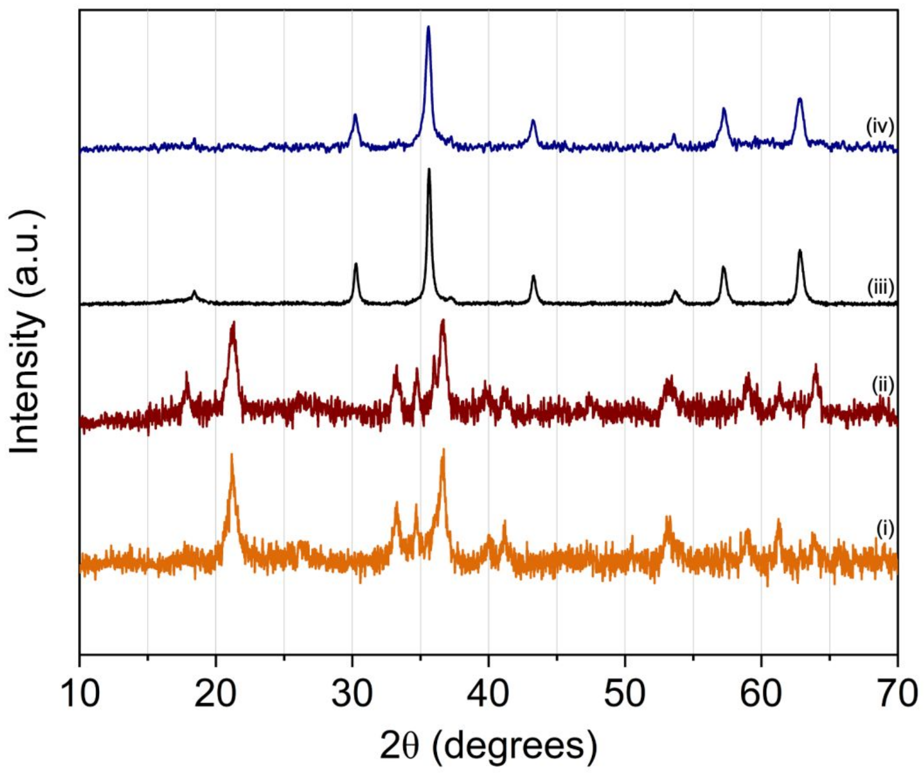

Figure S11. Powder X-ray diffraction patterns after N_2 sorption measurements. The patterns confirm that the phase composition of (i and iii) goethite and magnetite pre-degassing and (ii-iv) goethite and magnetite post-degassing remains unchanged following overnight activation at 90 °C under flowing N_2, with no evidence of oxidation or transformation.

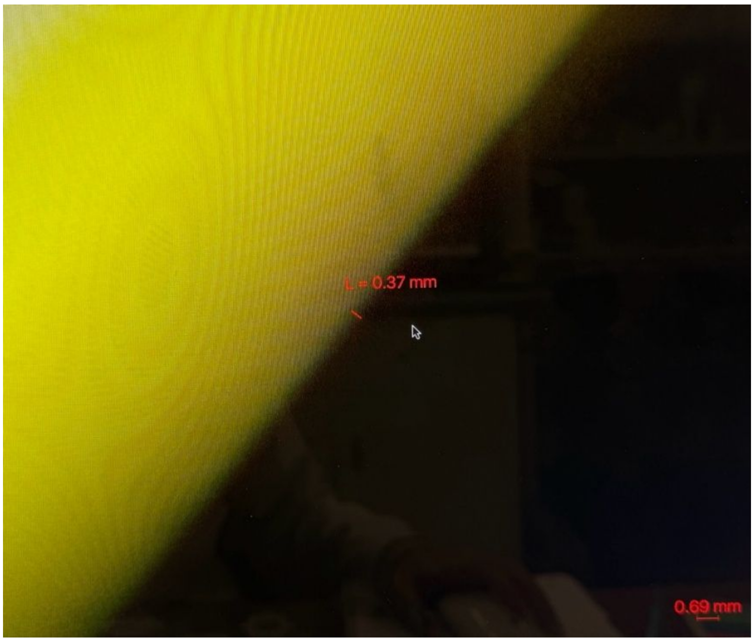

Figure S12. Optical micrograph of the reaction interface between the goethite (yellow) and green rust (green) regions. The red marker indicates a transition width of 0.37 mm, confirming the sharpness of the boundary relative to the macroscopic diffusion length.

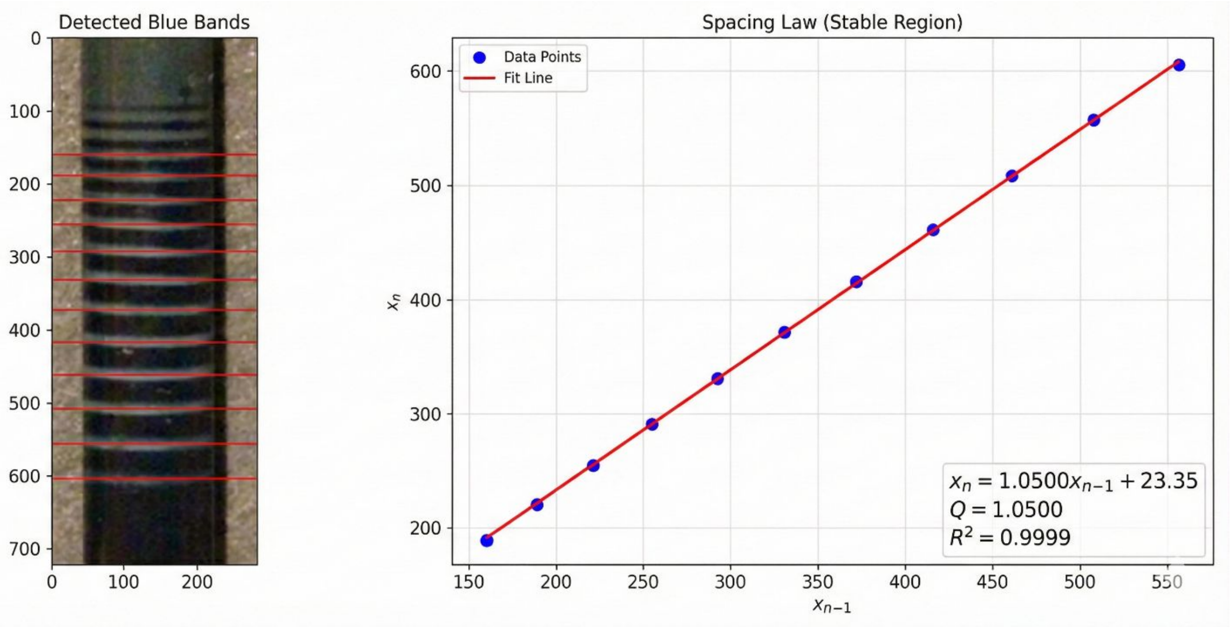

Figure S13. (Left) Digital extraction of periodic magnetite bands from the sample with [Fe^{2+}]:[Fe^{3+}] = 2.5:0.5. (Right) Jablczynski spacing law plot showing the position of the nth band x_n (in pixels) versus the preceding band (x_{n-1}). The linear fit yields a spacing coefficient (slope) of Q = 1.050 with $R^2 > 0.999$, confirming the regularity of the self-organized pattern.

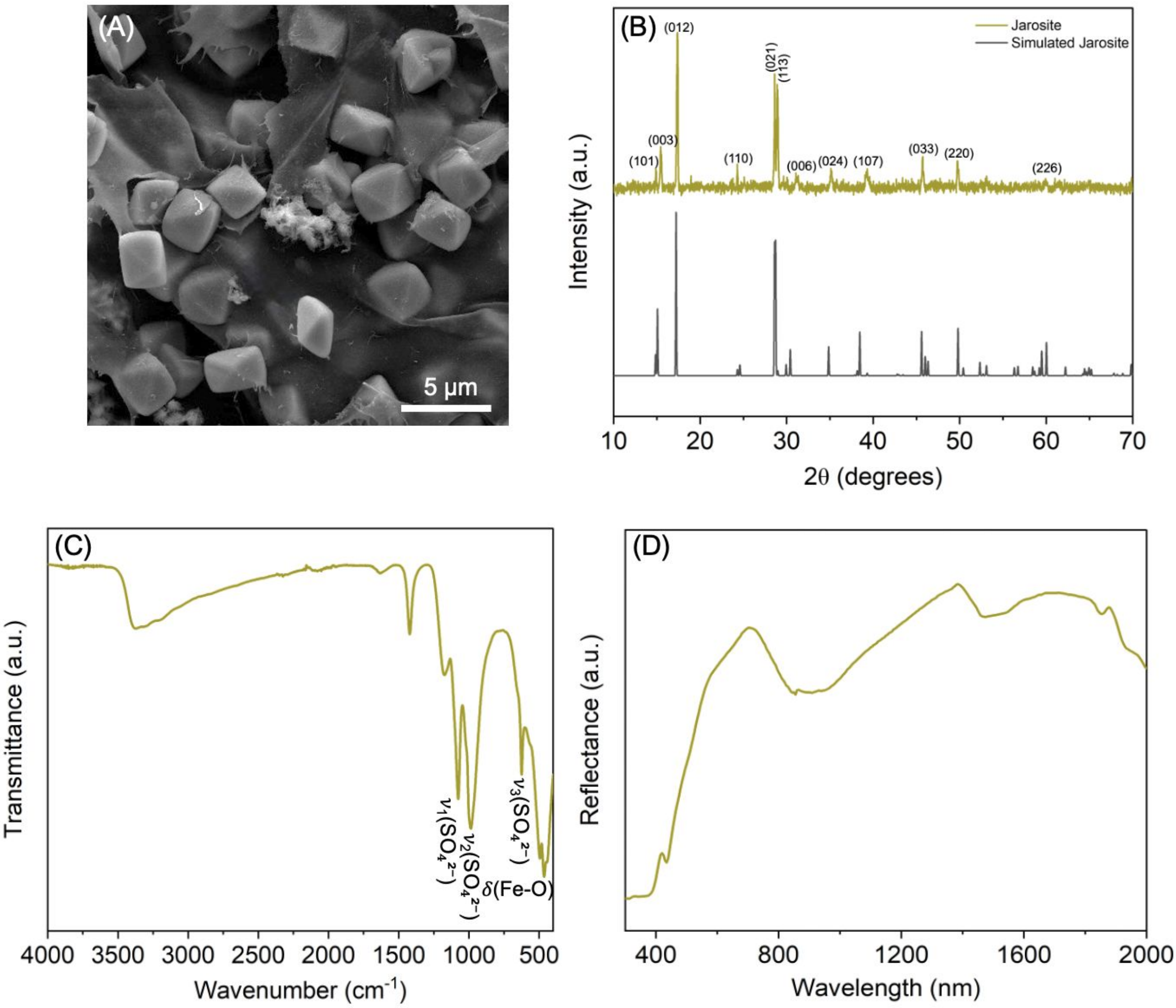

Figure S14. (A) Scanning electron microscopy (SEM) images of jarosite showing rhombohedral crystals obtained after a diffusion of 10 days; (B) PXRD pattern of jarosite; (C) ATR-FTIR transmittance spectra of jarosite exhibiting strong stretching vibrations of sulfate at (~1193 cm^{-1}, 623 cm^{-1} and ~1082 cm^{-1}) and Fe–O vibration around 500 cm^{-1}; (D) UV-Vis diffuse reflectance spectra of jarosite displaying a charge-transfer absorption band at 435 nm due to the effect of the Fe electronic absorption.

Table S1. Fitting tables of the diffusion profile at different iron concentrations of Goethite ($d_G = \alpha_G t^{\beta_G}$).

**$[Fe^{2+}]$/M:$[Fe^{3+}]$/M**	**α_G [mm $h^{-1/2}$]**	**± Δα**	**β_G**	**± Δβ**	**R^2**
0.20:0.40	1.79	0.01	0.464	0.001	1.000
0.15:0.30	2.02	0.01	0.476	0.001	1.000
0.10:0.20	2.06	0.02	0.504	0.002	0.999
0.07:0.14	1.91	0.03	0.541	0.003	0.998
0.04:0.08	1.99	0.09	0.560	0.008	0.994

Table S2. Fitting tables of the diffusion profile at different iron concentrations of Green rust ($d_{GR} = \alpha_{GR} t^{\beta_{GR}}$).

**$[Fe^{2+}]$/M:$[Fe^{3+}]$/M**	**α_{GR} [mm $h^{-1/2}$]**	**± Δα**	**β_{GR}**	**± Δβ**	**R^2**
0.20:0.40	1.73	0.02	0.463	0.002	0.999
0.15:0.30	2.01	0.02	0.470	0.002	0.999
0.10:0.20	2.32	0.02	0.472	0.002	0.999
0.07:0.14	2.57	0.04	0.472	0.003	0.997
0.04:0.08	2.79	0.16	0.477	0.010	0.975

Table S3. Fitting tables of the diffusion profile at different iron concentrations of Magnetite ($d_M = \alpha_M t^{\beta_M}$).

**$[Fe^{2+}]$/M:$[Fe^{3+}]$/M**	**α_M[mm $h^{-1/2}$]**	**± Δα**	**β_M**	**± Δβ**	**R^2**
0.20:0.40	1.91	0.06	0.400	0.006	0.988
0.15:0.30	1.96	0.05	0.436	0.005	0.992
0.10:0.20	1.96	0.04	0.469	0.004	0.995
0.07:0.14	2.57	0.04	0.472	0.003	0.987
0.04:0.08	2.83	0.19	0.439	0.013	0.965

Table S4. Fitting tables of the diffusion profile under varying hydroxide concentrations of Goethite ($d_G = \alpha_G t^{\beta_G}$).

Outer [OH⁻]/M	α_G [mm h$^{-1/2}$]	± Δα	β_G	± Δβ	R^2
1.0	0.24	0.03	0.391	0.009	0.974
1.5	0.24	0.03	0.440	0.006	0.990
2.0	0.24	0.03	0.437	0.005	0.994
2.5	0.32	0.04	0.425	0.005	0.993
3.0	0.35	0.04	0.406	0.004	0.994

Table S5. Fitting tables of the diffusion profile under varying hydroxide concentrations of Green rust ($d_{GR} = \alpha_{GR} t^{\beta_{GR}}$).

Outer [OH⁻]/M	α_{GR} [mm h$^{-1/2}$]	± Δα	β_{GR}	± Δβ	R^2
1.0	0.24	0.03	0.392	0.012	0.949
1.5	0.24	0.03	0.428	0.006	0.987
2.0	0.24	0.03	0.439	0.005	0.994
2.5	0.32	0.04	0.431	0.005	0.993
3.0	0.35	0.04	0.417	0.004	0.994

Table S6. Fitting tables of the diffusion profile under varying hydroxide concentrations of Magnetite ($d_M = \alpha_M t^{\beta_M}$).

Outer [OH⁻]/M	α_M[mm h$^{-1/2}$]	± Δα	β_M	± Δβ	R^2
1.0	0.24	0.03	0.466	0.005	0.994
1.5	0.24	0.03	0.471	0.004	0.995
2.0	0.24	0.03	0.479	0.004	0.996
2.5	0.32	0.04	0.490	0.005	0.995
3.0	0.35	0.04	0.539	0.005	0.996

Table S7. Summary of the textural and physicochemical properties of goethite, magnetite, and green rust from Nitrogen physisorption analysis, which reveals the distinct porosity evolution of the three phases.

Region	Isotherm Type	Hysteresis Loop Type	Surface Area ($m^2 \cdot g^{-1}$)	Total Pore Volume ($cm^3 \cdot g^{-1}$)	Avg. Pore Diameter (4V/A) (nm)
**Goethite**	IV	H3	116	0.244	8.4
**Green rust**	IV	H3	23	0.147	25.6
**Magnetite**	IV	H1	60	0.194	12.9

Video Captions

Video S1. Effect of Outer Hydroxide Concentration.

Time-lapse recording of the precipitation–diffusion process in 1.0 wt% agar hydrogels containing fixed inner iron concentrations ([Fe^{2+}] = 0.10 M, [Fe^{3+}] = 0.20 M).

Tube arrangement (Left to Right): Increasing outer NaOH concentration: 1.0 M, 1.5 M, 2.0 M, 2.5 M, and 3.0 M.

Note how the front velocity and region thickness increase systematically with higher hydroxide driving force.

Video S2. Effect of Inner Iron Concentration.

Time-lapse recording at fixed outer alkalinity ([NaOH] = 3.0 M) with varying total iron loading.

Tube arrangement (Left to Right): Decreasing total iron concentration ([Fe^{2+}] :([Fe^{3+}]): 0.20:0.40 M, 0.15:0.30 M, 0.10:0.20 M, 0.07:0.14 M, and 0.04:0.08 M.

Note that fronts propagate faster in the tubes with lower iron content (Right) due to the reduced local alkalinity demand (Λ).

Video S3. Effect of Fe(II)/Fe(III) Ratio (Transition to Liesegang Banding).

Time-lapse recording at fixed outer alkalinity ([NaOH] = 3.0 M) and fixed total iron (0.30 M), varying the oxidation state ratio.

Tube arrangement (Left to Right): Increasing Fe(II) content ([Fe^{2+}] :([Fe^{3+}]): 2.0:1.0, 2.25:0.75, 2.5:0.5, 2.75:0.25, and 3.0:0.

Observe the transition from steady continuous fronts (Left) to the onset of periodic Liesegang banding (Right) as the system becomes enriched in Fe(II), increasing the sink strength and creating supersaturation–depletion cycles.
